# Characterization of an *Aspergillus niger* for Efficient Fatty Acid Ethyl Ester Synthesis in Aqueous Phase and the Molecular Mechanism

**DOI:** 10.3389/fmicb.2021.820380

**Published:** 2022-02-21

**Authors:** Youqiang Xu, Huiqin Huang, Hongyun Lu, Mengqin Wu, Mengwei Lin, Chunsheng Zhang, Zhigang Zhao, Weiwei Li, Chengnan Zhang, Xiuting Li, Baoguo Sun

**Affiliations:** ^1^Beijing Advanced Innovation Center for Food Nutrition and Human Health, Beijing Technology and Business University, Beijing, China; ^2^Beijing Engineering and Technology Research Center of Food Additives, Beijing Technology and Business University, Beijing, China; ^3^Chengde Qianlongzui Distillery Company, Hebei, China; ^4^Key Laboratory of Brewing Molecular Engineering of China Light Industry, Beijing Technology and Business University, Beijing, China

**Keywords:** strong-flavor baijiu, fatty acid ethyl ester, *Aspergillus niger*, enzyme, aqueous phase, molecular mechanism

## Abstract

Fatty acid ethyl esters are important flavor chemicals in strong-flavor baijiu. Microorganisms are the main contributors to ester synthesis during baijiu manufacture. However, the ester synthesis was unstable between batches. This was owing to a limited knowledge of the mechanisms for ester synthesis by microorganisms. In this work, a fatty acid ethyl ester synthesizing *Aspergillus niger* strain CGMCC (China General Microbiological Culture Collection) 3.4309 was identified. The conversion ratios of ethyl valerate, ethyl caproate, ethyl caprylate, and ethyl caprate were 7.87, 29.20, 94.80, and 85.20%, respectively, under the optimized conditions. A comparison of transcriptomes under the initial and optimized ester synthetic conditions indicated that 23 genes were upregulated in transcription level and encoded enzymes with potential abilities for ester synthesis. Eleven of the enzymes were expressed, and three of them, numbered An605, An1097, and An3131, showed the ability to catalyze fatty acid ethyl ester synthesis under aqueous phase, with capric acid as the preferred substrate. The possible enzymatic catalytic mechanism was proposed based on homology modeling and molecular docking. This study reported for the first time that *A. niger* showed the ability to efficiently catalyze the synthesis of short- and medium-chain fatty acid ethyl esters in aqueous phase, identified the key enzymes, and analyzed the basic enzymatic properties. This is helpful to promote the application of related microorganisms and enzyme resources in the baijiu industry.

## Introduction

Chinese traditional fermented baijiu is an important composition of Chinese food (Xu et al., 2022b). Strong-flavor baijiu accounts for more than 70% of the baijiu market share and is a representative of traditional fermented alcoholic beverages (Xu et al., 2021b). Although with a history of thousands of years, strong-flavor baijiu faces problems in the process of modernization. The manufacturing processes are still based on an empirical mode, with low efficiency and poor stability between batches (Xu et al., 2022b). The ultimate reason is that the core functional microorganisms and the formation mechanisms of the flavor components are still not yet clear, although some studies reported about baijiu fermentation (Hong et al., 2021; Wu et al., 2021; Xu et al., 2021b). Therefore, it is urgent to clarify the mechanism of synthesizing flavor substances in traditional strong-flavor baijiu, so as to ensure the quality of products. This is an inevitable requirement for the modernization of the baijiu industry (Ye et al., 2021).

Esters are important flavor compounds in baijiu (Xu et al., 2022b). The national standard stipulates that strong-flavor baijiu possesses a mixed flavor with the flavor of ethyl caproate as the principal aroma components, indicating that ethyl esters, especially ethyl caproate, are important contributors to the flavor of the product (Xu et al., 2017). Ethyl caproate endows the product with a cellar fragrance and is recognized as a characteristic of high-quality strong-flavor baijiu (Wei et al., 2020). Other esters such as ethyl acetate, ethyl lactate, and ethyl butyrate are also important esters with fruity and sweet flavors (Xu et al., 2022b). Studies show that microbial metabolism is important for synthesizing fatty acid ethyl esters, including four known microbial synthetic pathways (Kruis et al., 2019). The first route is the synthesis of ethyl acetate catalyzed by alcohol acyltransferases using alcohol and acyl-CoA as the substrates (Kruis et al., 2017). The second one is the synthesis of esters catalyzed by Baeyer–Villiger monooxygenases using 2-ketone as the substrate coupled with the consumption of oxygen and NAD(P)H (Tang et al., 2013). The third one converts alcohol and aldehyde to the corresponding ethyl ester coupled with the co-factor NAD(P)^+^ through hemiacetal dehydrogenation (Yurimoto et al., 2005). The fourth one catalyzes the esterification reaction by the esterifying enzymes such as lipase and esterase using alcohols and acids as the substrates (Xu et al., 2021b). Baijiu flavor analysis indicates that only a slight amount of aldehyde and ketone is produced during the anaerobic fermentation process; thus, the second and third routes do not play a major role for the esterification of ethyl esters (Xu et al., 2022b). The first route is critical to the synthesis of ethyl acetate (Kruis et al., 2017). However, the ratios between ethyl esters have a great influence on the baijiu flavor (Xu et al., 2022b). For strong-flavor baijiu, the concentration of ethyl caproate is significantly higher than that of ethyl acetate (Xu et al., 2017). If ethyl caproate is mainly synthesized *via* alcohol and the acyl-CoA pathway, a large amount of ethyl acetate would be generated as the byproduct. It will lead to the improper ratio of ethyl caproate to ethyl acetate and affects the flavor-style of strong-flavor baijiu. Therefore, the preferred pathway to synthesize fatty acid ethyl esters of strong-flavor baijiu, especially ethyl caproate, is through the esterification reaction of fatty acids and ethanol. This has also been verified by the fact that the strength of caproic acid-producing strains during fermentation is helpful for the stable synthesis of ethyl caproate (Zou et al., 2018). The main metabolite of caproic acid-producing strains is medium-chain fatty acids dominated by caproic acid and provides a sufficient substrate for esterification (Zou et al., 2018).

Although microorganisms are recognized to play an important role in fatty acid ethyl ester synthesis during baijiu fermentation, studies about the related microorganisms are still scarce. Taking strong-flavor baijiu as an example, many microorganisms are involved in the fermentation process, while only *Burkholderia pyrrocinia*, *Clavispora lusitaniae*, *Lichtheimia ramosa*, and *Monascus purpureus* were reported with the ability to synthesize fatty acid ethyl esters (Chung et al., 2017; Fan et al., 2020, 2021; Xu et al., 2021b). In addition, although some of these microorganisms promoted ester synthesis in practical application, bottleneck problems existed such as unstable ester concentrations among batches and unclear technical conditions for the actual application of these species (Xu et al., 2022b). The reason is that little is known about the characteristics of the key enzymes that catalyze fatty acid ethyl ester synthesis from microorganisms. Strong-flavor baijiu is mainly produced by solid-state fermentation. Grains [mainly sorghum (*Sorghum bicolor* L.)] are used as raw materials, and the water content of fermented grains is between 53 and 58% and can be recognized as an aqueous-phase system (Xu et al., 2017). The best esterification condition of the known enzymes is the organic-phase system (Bollinger et al., 2020). In the aqueous-phase system, these enzymes mainly show the properties for hydrolyzing esters and serve as one of the reasons for the unstable fatty acid ethyl ester production (Ferrer et al., 2015; Bollinger et al., 2020).

In this work, *Aspergillus niger* from baijiu showed prominent ability for ester synthesis in aqueous phase. For example, under the optimized culture conditions, the conversion ratio of ethyl caprylate was 94.80% in aqueous phase by *A. niger* CGMCC (China General Microbiological Culture Collection) 3.4309. Therefore, it was reasonable to conclude that the *A. niger* CGMCC 3.4309 carried the enzyme for catalyzing fatty acid ethyl ester synthesis in an aqueous-phase system. However, studies on ester synthesis by *A. niger* species were focused on the organic-phase system so far and was obviously different from the aqueous-phase system (Bourne et al., 2004; Mhetras et al., 2010; Cong et al., 2019). The enzyme responsible for catalyzing ester synthesis in the aqueous-phase system was rarely reported. Therefore, the enzymes catalyzing ester synthesis were identified and investigated from *A. niger* CGMCC 3.4309 in the aqueous-phase system, and the possible catalytic mechanism was proposed. This will help to reveal the molecular mechanism of *A. niger* for fatty acid ethyl ester synthesis in the aqueous-phase system and promotes the application of the strain for baijiu fermentation.

## Materials and Methods

### Materials and Media

Acetic acid was purchased from Beijing Mairuida Technology Co., Ltd. Propionic acid was purchased from Alfa aesar (China) Chemical Co., Ltd. Butyric acid and caproic acid were purchased from Shanghai Aladdin Biochemical Technology Co., Ltd. Valeric acid was purchased from Beijing J&K Co., Ltd. Caprylic acid was purchased from Shanghai McLean Biochemical Technology Co., Ltd. Capric acid was purchased from Damas Beta Company. Soluble starch was purchased from Xilong Science Co., Ltd. Sucrose, maltose, and glucose were purchased from Fuchen (Tianjin) Chemical Reagent Co., Ltd. Sorghum powder was purchased from Tianjin Lifalong Chemical Technology Co., Ltd. Soybean peptone, tryptone, yeast extract, lactose, and beef extract were purchased from Beijing Aoboxing Biotechnology Co., Ltd. Corn pulp dry powder was purchased from Yuanye Biotechnology Co., Ltd. The potato dextrose agar (PDA) medium contained glucose (20 g/L), potato (200 g/L), and agar powder (20 g/L) with natural pH value. Fermentation medium contained glucose (100 g/L), soybean powder (10 g/L), ammonium chloride (12 g/L), sodium dihydrogen phosphate (1 g/L), and magnesium sulfate (0.2 g/L) with pH 4.5. All other chemicals were analytical-grade reagents and commercially available.

### Strain Selection

A total of 10 strains of *A. niger* deposited in CGMCC were investigated for ester synthesis in aqueous phase after being cultured by a fermentation medium for 7 days. Single-factor optimization was carried out to investigate the various factors affecting the production of enzymes for esterification. The strain was cultured on the PDA plate and then transferred to the fermentation medium. It was cultured in a 250 ml flask containing 100 ml fermentation medium at 28°C and 200 r/min for 7 days. The cell culture (30 ml) was transferred into a 50 ml tube for ultrasonic cell crushing. The crushed cell solution was centrifuged at 4°C and 9,000 × *g* for 5 min.

The supernatant was used as the crude enzyme solution to determine the ester synthetic ability. The reaction mixture contained crude enzyme solution (2 ml), mixed fatty acids (acetic acid, propionic acid, butyric acid, valeric acid, caproic acid, caprylic acid, and capric acid, 10 mM for each substrate), ethanol (1 M), and a citric acid buffer (50 mM, pH 4.0) to the final volume of 10 ml. The reaction mixture was kept in a water bath at 30°C and 200 r/min for 12 h. Thereafter, *n*-hexane (3 ml) was added to the reaction system and vortexed for 30 s. The mixture was centrifuged for 10 min at 4°C and 13,000 × *g*, and the upper-layer *n*-hexane was filtered using a 0.22 μm membrane and used for gas chromatography (GC) analysis.

### Optimization of Fermentation Conditions

#### Optimization of Nitrogen and Carbon Sources

The six carbon sources were glucose, sucrose, soluble starch, maltose, lactose, and sorghum powder. The seven nitrogen sources were corn slurry dry powder, soybean peptone, yeast extract, beef extract, NH_4_NO_3_, (NH_4_)_2_SO_4_, and NH_4_Cl. *A. niger* was cultured using the fermentation medium containing the respective carbon or nitrogen sources in flasks, and kept at 28°C and 200 r/min for 7 days, and used for analysis. The conversion ratios of fatty acids were calculated, and the optimal carbon and nitrogen sources were selected according to the conversion ratios of the substrates.

#### Optimization of Culture Temperature and Rotation Rates

After inoculation, the cell cultures were kept at 200 r/min under different temperatures (20, 25, 28, 30, 37, and 42°C) for 7 days. The culture medium inoculated with *A. niger* was kept at 28°C for 7 days at different rotation rates (50, 100, 150, 200, 250, and 300 r/min, respectively). The optimal culture temperature and rotation rates were determined according to the conversion ratios of the substrates.

### Transcriptome Analysis

After the medium composition and culture condition optimization, *A. niger* CGMCC 3.4309 was cultured under optimized and initial conditions, respectively. Total RNA samples were extracted, respectively, for transcriptome sequencing. Samples were prepared according to the standard protocol by the BGI Co. (Beijing, China). The quality of the sequenced reads was controlled after filtering out the reads with low quality, joint pollution, and unknown bases. Trinity was used to the *de novo* assembly of clean reads, and then, Tgicl was used to cluster the assembled transcriptome data to remove redundancy and generated unigenes (Supplementary Table 1; Pertea et al., 2003). Thereafter, functional annotation was performed of unigenes. The unigene transcription levels of each sample were calculated and compared. Genes with differentially transcription levels were analyzed by in-depth cluster analysis and functional enrichment analysis. Seven public databases were used for sequence comparison, including non-redundant protein sequence database (NR), nuclear sequence database (NT), Pfam, clusters of orthogonal groups (COGs) for eukaryotic complete genes, the Swiss prot protein sequence database (Swiss prot) and Kyoto Encyclopedia of Genes and Genomes (KEGG), and Gene Ontology (GO). The gene transcription levels were analyzed by using Poisson distribution. The genes with differentiated transcription levels were identified by a strict algorithm between samples. If the number of reads corresponding to gene numbered A is *x* in a large gene library, the expression of each gene accounts for only a small part of all genes. The distribution of *x* follows Poisson distribution (Equation 1). Among them, λ is the transcriptional copy number determined of gene A (Audic and Claverie, 1997; Li and Dewey, 2011).


(1) $$p\,\left(\text{x}\right)=\frac{e^{-\lambda }\,\lambda^{x}}{x^{!}}$$

The total number of reads uniquely aligned to the genome in sample 1 is N_1_. The total number of reads uniquely aligned to the genome in sample 2 is N_2_. If the total reads uniquely matched to gene A in sample 1 is *x*, and in sample 2 is *y*, then the relative transcription divergence of gene A in the two samples can be calculated by the following equation (Equation 2) (Audic and Claverie, 1997; Li and Dewey, 2011). Differentially transcripted genes were identified as genes with varied transcription levels of more than twofold.


$$2\,\sum_{i=0}^{i=y}p\,\left(i/x\right)$$

or $2\times (1-\sum_{i=0}^{i=y}p\left(i/x\right)$ if $\sum_{i=0}^{i=y}p\,\left(i/x\right)>0.5$


(2) $$p\,\left(\frac{\text{y}}{\text{x}}\right)={\left(\frac{N_{2}}{N_{1}}\right)}^{y}\,\frac{{\left(x+y\right)}^{!}}{x^{!}\,y^{!}\,{\left(1+\frac{N_{2}}{N_{1}}\right)}^{\left(x+y+1\right)}}$$

### Expression and Identification of Potential Enzymes for Esterification

#### Gene Library Construction

The linearized vector pET-28a(+) was generated by PCR amplification using the primer pair pET28a.f/pET28a.r (Table 1). Genes were amplified through PCR with complementary DNA (cDNA) as the template from the reverse transcription of the total RNA sample using the primer pairs listed in Table 1. The 15–20 bp homologous recombinant sequence regions to the linearized vector pET-28a(+) were added to the 5′- and 3′-regions of the genes, respectively. Plasmids pET-An408, pET-An605, pET-An923, pET-An1097, pET-An1486, pET-An2017, pET-An2076, pET-An2794, pET-An3040-3, pET-An3131, pET-An3196, pET-An3559, pET-An3936, pET-An5004, and pET-An5100 were constructed according to the protocol of the Vazyme ClonExpress II One Step Cloning Kit (Vazyme Biotech, Nanjing, China). All the constructed plasmids were transferred into *E. coli* BL21(DE3) to generate the respective transformants as listed in Table 1.

**TABLE 1:** Strains, vectors, and primers used in this work.

Strain	Characteristic	References
*Escherichia coli* BL21(DE3)	Used as host strain	Invitrogen
*E. coli* BL21/pET-28a(+)	*E. coli* BL21(DE3) harboring expression vector pET-28a(+)	This work
*E. coli* BL21/pET-An408	*E. coli* BL21(DE3) harboring expression vector pET-An408	This work
*E. coli* BL21/pET-An605	*E. coli* BL21(DE3) harboring expression vector pET-An605	This work
*E. coli* BL21/pET-An923	*E. coli* BL21(DE3) harboring expression vector pET-An923	This work
*E. coli* BL21/pET-An1097	*E. coli* BL21(DE3) harboring expression vector pET-An1097	This work
*E. coli* BL21/pET-An1486	*E. coli* BL21(DE3) harboring expression vector pET-An1486	This work
*E. coli* BL21/pET-An2017	*E. coli* BL21(DE3) harboring expression vector pET-An2017	This work
*E. coli* BL21/pET-An2076	*E. coli* BL21(DE3) harboring expression vector pET-An2076	This work
*E. coli* BL21/pET-An2794	*E. coli* BL21(DE3) harboring expression vector pET-An2794	This work
*E. coli* BL21/pET-An3040-3	*E. coli* BL21(DE3) harboring expression vector pET-An3040-3	This work
*E. coli* BL21/pET-An3131	*E. coli* BL21(DE3) harboring expression vector pET-An3131	This work
*E. coli* BL21/pET-An3196	*E. coli* BL21(DE3) harboring expression vector pET-An3196	This work
*E. coli* BL21/pET-An3559	*E. coli* BL21(DE3) harboring expression vector pET-An3559	This work
*E. coli* BL21/pET-An3936	*E. coli* BL21(DE3) harboring expression vector pET-An3936	This work
*E. coli* BL21/pET-An5004	*E. coli* BL21(DE3) harboring expression vector pET-An5004	This work
*E. coli* BL21/pET-An5100	*E. coli* BL21(DE3) harboring expression vector pET-An5100	This work

**Vector**	**Characteristic**	**References**

pET-28a(+)	pMB1 replicon, Kanr, carried PT7 promoter	Novagen
pET-An408	pET-28a(+) inserted with the gene numbered An408	This work
pET-An605	pET-28a(+) inserted with the gene numbered An605	This work
pET-An923	pET-28a(+) inserted with the gene numbered An923	This work
pET-An1097	pET-28a(+) inserted with the gene numbered An1097	This work
pET-An1486	pET-28a(+) inserted with the gene numbered An1486	This work
pET-An2017	pET-28a(+) inserted with the gene numbered An2017	This work
pET-An2076	pET-28a(+) inserted with the gene numbered An2076	This work
pET-An2794	pET-28a(+) inserted with the gene numbered An2794	This work
pET-An3040-3	pET-28a(+) inserted with the gene numbered An3040-3	This work
pET-An3131	pET-28a(+) inserted with the gene numbered An3131	This work
pET-An3196	pET-28a(+) inserted with the gene numbered An3196	This work
pET-An3559	pET-28a(+) inserted with the gene numbered An3559	This work
pET-An3936	pET-28a(+) inserted with the gene numbered An3936	This work
pET-An5004	pET-28a(+) inserted with the gene numbered An5004	This work
pET-An5100	pET-28a(+) inserted with the gene numbered An5100	This work

**Primer name^a^**	**Sequence (5**′**-3**′**)*^b^***	

pET28a.f	ATCGCACTCGAGCACCACC	
pET28a.r	CATGGTATATCTCCTTCTTA	
An408.f	TAAGAAGGAGATATACCATGATGCTTGGCCGCTTGGAG	
An408.r	GGTGGTGCTCGAGTGCGATATCCCATCGAGTGAAATA	
An605.f	TAAGAAGGAGATATACCATGATGCATACTCCATATCTTC	
An605.r	GGTGGTGCTCGAGTGCGATGTAGCTCCGATCAATCCAG	
An923.f	TAAGAAGGAGATATACCATGATGAAGAAGACGCTGCTC	
An923.r	GGTGGTGCTCGAGTGCGATCTCCACGACCACAACGTC	
An1097.f	TAAGAAGGAGATATACCATGATGCTAAAGCTCGCTGTTG	
An1097.r	GGTGGTGCTCGAGTGCGATTCGCCAGTACGCGTTGGCC	
An1486.f	TAAGAAGGAGATATACCATGATGCATTTTTCTCATTCT	
An1486.r	GGTGGTGCTCGAGTGCGATCACCCCCAACTCACTCGC	
An2017.f	TAAGAAGGAGATATACCATGATGCTAGACGTGTCACTTG	
An2017.r	GGTGGTGCTCGAGTGCGATCATCATCCAAACCCCAAC	
An2076.f	TAAGAAGGAGATATACCATGATGACCGCGTATGGCGGC
An2076.r	GGTGGTGCTCGAGTGCGATAATCTCCATTTCCGGCGC
An2794.f	TAAGAAGGAGATATACCATGATGAAGTCAACTACACCT
An2794.r	GGTGGTGCTCGAGTGCGATGTAAAGAGTACCGCAGTAC
An3040-3.f	TAAGAAGGAGATATACCATGATGGATTACTCCGTCAAC
An3040-3.r	GGTGGTGCTCGAGTGCGATTGACCGTACATGGTAACT
An3131.f	TAAGAAGGAGATATACCATGATGTCTGCGCTTAAAGAC
An3131.r	GGTGGTGCTCGAGTGCGATCCCTGTTAGTTCCCGTTTC
An3196.f	TAAGAAGGAGATATACCATGATGGGGATAAAGCCAAAG
An3196.r	GGTGGTGCTCGAGTGCGATAGGCGTCCTAGCACCTTC
An3559.f	TAAGAAGGAGATATACCATGATGGCCACTCTTAGAATG
An3559.r	GGTGGTGCTCGAGTGCGATTACTCCGGCCTCTGCTCG
An3936.f	TAAGAAGGAGATATACCATGATGCAGCTTCAATTCATC
An3936.r	GGTGGTGCTCGAGTGCGATGACAGTAAGATTGGCTC
An5004.f	TAAGAAGGAGATATACCATGATGATTCGGCTTTTGCTAC
An5004.r	GGTGGTGCTCGAGTGCGATGGCAGAATGTACAAAAGT
An5100.f	TAAGAAGGAGATATACCATGATGGCGTCCCACTATCTC
An5100.r	GGTGGTGCTCGAGTGCGATTTCAACAATCGAACGAATC
An163-2.f	TAAGAAGGAGATATACCATGATGCGGCTCGCGCCCCAG
An163-2.r	GGTGGTGCTCGAGTGCGAT GGGAAAAGGACAGGTTG
An163-6.f	TAAGAAGGAGATATACCATGATGGCCTACATGCTGTAC
An163-6.r	GGTGGTGCTCGAGTGCGATTTCGTAAGTAACCTGGGC
An286.f	TAAGAAGGAGATATACCATGATGATCGCTCCCAAAATC
An286.r	GGTGGTGCTCGAGTGCGATCAAAGTCTCCCCCAACCA
An1556-4.f	TAAGAAGGAGATATACCATGATGAAGCCCGGGTATTGC
An1556-4.r	GGTGGTGCTCGAGTGCGATTTGAGGTACCCTCTCCGA
An2505.f	TAAGAAGGAGATATACCATGATGAAGGATGATTACAAC
An2505.r	GGTGGTGCTCGAGTGCGATATAAGGCACATACTCCTC
An3040-1.f	TAAGAAGGAGATATACCATGATGGCAGATGGCGGCGGC
An3040-1.r	GGTGGTGCTCGAGTGCGATTGACCGCACATGGTAAC
An2588.f	TAAGAAGGAGATATACCATGATGGGATCGCAGGCCGCG
An2588.r	GGTGGTGCTCGAGTGCGATGTAAGTCTTCAGTGTGTC
An2763.f	TAAGAAGGAGATATACCATGATGGGGTCCTGGACGACC
An2763.r	GGTGGTGCTCGAGTGCGATATAATTCGAATCGTCCTG

*^a^.f means sense prime, .r means antisense prime.*

*^b^Underlined region is the homologous recombinant sequence to the ends of 5′- or 3′-terminal of the linearized pET-28a(+) vector.*

#### Inducement of Enzyme Expression

The transformants were inoculated, respectively, to the Lysogeny Broth (LB) medium (added with kanamycin to a final concentration of 40 μg/ml), and kept at 37°C with stirring at 200 r/min for about 3 h. When the cell density reached the absorbance of 0.6–0.8 at OD_600nm_, isopropyl β-D-1-thiogalactopyranoside (IPTG) was added to a final concentration of 0.5 μM, and kept at 25°C with stirring at 200 r/min for about 20 h. The cell culture was centrifuged at 13,000 × *g* for 5 min at 4°C, and washed twice with a Tris-HCl buffer (50 mM, pH 7.0). Cells were finally suspended by the Tris-HCl buffer and disrupted with a sonifier (Ningbo Xinzhi, Ningbo, China), and centrifuged thereafter. The supernatant was used as the crude enzyme solution for analysis. The crude enzyme solution was analyzed by sodium dodecyl sulfate - polyacrylamide gel electrophoresis (SDS-PAGE) to verify enzyme expression.

#### Esterification Using Whole Cell Catalysis

Whole cell catalysis was used for enzymatic esterification analysis. After the addition of IPTG and being cultured for 20 h, Triton X-100 was added to the cell culture to the final concentration of 0.3% (v/v). Ethanol was added to the final concentration of 5.0 g/L, and valeric acid, caproic acid, caprylic acid, and capric acid were added to the final concentration of 10 mM for each chemical, and kept at 28°C and 250 r/min for 8 h. *n*-Hexane was added to the reaction mixture by 10 ml, vortexed for 30 s, and transferred to a 50 ml tube for centrifugation at 4°C and 13,000 × *g* for 10 min. The upper layer *n*-hexane was filtered using a 0.22 μm membrane and used for GC analysis.

#### Enzyme Purification and Property Analysis

The crude enzyme solution was purified using an Ni Sepharose high performance (HP) column (1 ml; GE, Uppsala, Sweden) with a phosphate buffer (50 mM, pH 7.4, 400 mM NaCl) and varied concentrations of imidazole using ÄKTA Fast Protein Liquid Chromatography purification system (GE, Uppsala, Sweden). The purified enzyme was confirmed by SDS-PAGE and quantified by Coomassie brilliant blue dyeing method using bovine serum albumin as the standard.

The enzyme was used for esterification with ethanol and fatty acids as the substrates. The reaction system contained the known amount of purified enzyme solution, capric acid (a final concentration of 10 mM), ethanol (1 M), and reaction buffer to a final volume of 1 ml. The effect of pH on enzyme activity was analyzed with pH values from 3.0 to 8.0 (Na_2_HPO_4_/citric acid buffer, 50 mM) at 30°C. For pH stability analysis, the enzyme solution was added to an Na_2_HPO_4_/citric acid buffer (50 mM, pH 4.0), and kept for 0.5 or 1.0 h. Thereafter, the reaction mixture was prepared and kept at 30°C for 1.0 h. The optimum temperature was analyzed after the reaction mixture was kept at different temperatures in an Na_2_HPO_4_/citric acid buffer (50 mM, pH 4.0). For temperature stability analysis, the enzyme solution was kept at 30°C for 0.5 or 1.0 h. Thereafter, the reaction mixture was prepared in an Na_2_HPO_4_/citric acid buffer (50 mM, pH 4.0), and kept for 1.0 h. When detecting the effects of ions or surfactants on enzyme activity, a 2 mM ion or surfactant was added to the reaction mixture containing capric acid (10 mM) and ethanol (1 M), and kept at 30°C for 1 h.

### Homology Modeling and Molecular Docking

Homology modeling was performed using Discovery Studio Modeler V2019 software. The appropriate reference enzymes were selected from the PDB_nr95 database according to the sequence similarity. The secondary structure of the enzyme was predicted based on the sequence alignment with the reference enzymes. Thereafter, the three-dimensional (3D) structure model was constructed and the quality of the model was evaluated using Ramachandran plot analysis. The enzyme was set as the receptor and used for molecular docking. All the ligands (caproic acid, caprylic acid, capric acid, ethyl caproate, ethyl caprylate, and ethyl caprate) were downloaded from National Center for Biotechnology Information (NCBI) PubChem Compound.

Reference models were searched by similarities using the sequences of proteins as the templates in the Protein Data Bank (PDB) database. Sequence alignment was analyzed for finding the catalytic active sites. Molecular docking was carried out using Discovery Studio CDOCKER (V2019). The grid option tool was opened under the command of grid box to modify the receptor protein, including hydrogenation and charge balance. The size of the ligand binding pocket of the receptor enzyme was jointly determined and set as the center of the binding site. By calculating the equivalent comprehensive scores of the repulsion, hydrogen bond, hydrophobic interaction, and molecular flexibility of the receptor substrate complex, the affinity of the ligand and receptor was evaluated, and outputted in the format of the binding energy score. The binding energy was the core parameter to measure whether the substrate could effectively bind to the receptor molecule. A higher affinity of the ligand and receptor was usually related to a lower binding energy score (Williams et al., 2004). The proteins with sequence similarity lower than 25% were modeled using the online version of RoseTTAFold^1^ (Baek et al., 2021). Then, the Dali server^2^ (Holm and Laakso, 2016) was used to compare newly generated 3D models against structures in the PDB.

### Analytical Methods

The concentrations of the fatty acid esters were quantified by Agilent 7890B GC (Agilent, Santa Clara, CA, United States) equipped with a 19091N-213I column (30 m × 0.32 mm × 0.50 μm; Agilent, Santa Clara, CA, United States), a flame ionization detector, and an autosampler. The standard chemicals were serial-diluted and detected in triplicate to generate the standard curve. The GC analysis used an oven temperature program: 40°C for 5 min, increased to 170°C at 8°C/min, maintained for 10 min, increased to 240°C at 8°C/min, and maintained for 5 min. The carrier gas was nitrogen with a flow rate of 1.0 ml/min. The injection volume was 1.0 μl and was delivered in splitless mode. Origin 8.0 software was used for statistical analysis.

## Results and Discussion

### Identification and Optimization of *Aspergillus niger* Species Synthesizing Fatty Acid Ethyl Esters

**FIGURE 1:**
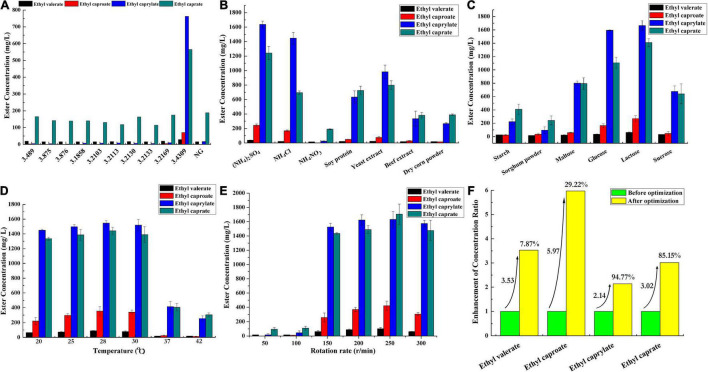
Identification and optimization of cultural conditions of *A. niger* for fatty acid ethyl ester synthesis. **(A)** Comparison of the esterification properties of different *A. niger* strains, NG, negative control, the medium without inoculation of *A. niger*. **(B)** Nitrogen source optimization for culturing *A. niger* CGMCC 3.4309. **(C)** Carbon source optimization for culturing *A. niger* CGMCC 3.4309. **(D)** Temperature optimization for culturing *A. niger* CGMCC 3.4309. **(E)** Rotation rate optimization for culturing *A. niger* CGMCC 3.4309. **(F)** The conversion rate comparison of the fatty acid ethyl esters by *A. niger* CGMCC 3.4309 before and after optimization.

Microorganisms are one of the deciding factors affecting baijiu quality (Jin et al., 2017; Wang et al., 2020, 2021). They not only metabolically synthesize flavor chemicals but also degrade some of the harmful chemicals in the raw materials, thus contributing greatly to the flavor and quality of the products (Xu Y. Q. et al., 2020; Xu et al., 2021a, 2022a). *A*. *niger* was generally recognized as an important functional microorganism in baijiu manufacture (Wu et al., 2015). Previous studies indicated its performance on the liquefying and saccharifying of the raw materials in the baijiu fermentation process (Wu et al., 2015; Jain and Katyal, 2018). Scarcely any studies investigated its function on esterification in an aqueous-phase system. In this work, the species also showed the esterification ability (Figure 1). *A. niger* species numbered CGMCC 3.489, CGMCC 3.1858, CGMCC 3.2169, CGMCC 3.2103, CGMCC 3.4309, CGMCC 3.2113, CGMCC 3.2130, CGMCC 3.0875, CGMCC 3.2133, and CGMCC 3.0876 were investigated, and the strain numbered CGMCC 3.4309 showed a superior esterification property. The conversion ratios of ethyl valerate, ethyl caproate, ethyl caprylate, and ethyl caprate were 2.23, 4.89, 44.30, and 28.20% (Figure 1), respectively.

Single-factor experiments were used to investigate the fermentation conditions and medium components that might affect the ester synthesis of *A*. *niger* CGMCC 3.4309, such as the type of carbon and nitrogen sources, fermentation temperature, and rotation rate. Seven nitrogen sources were analyzed including NH_4_Cl, soybean peptone, yeast extract, beef extract, NH_4_NO_3_, (NH_4_)_2_SO_4_, and corn slurry dry powder (Figure 1B). (NH_4_)_2_SO_4_ was the preferred nitrogen source. The conversion ratios of ethyl valerate, ethyl caproate, ethyl caprylate, and ethyl caprate were 2.94, 16.90, 95.00, and 62.00% (Figure 1B), respectively. Six kinds of carbon sources were investigated, including glucose, sucrose, soluble starch, maltose, lactose, and sorghum powder. Lactose served as the preferred carbon source, and the conversion ratios of ethyl valerate, ethyl caproate, ethyl caprylate, and ethyl caprate were 4.74, 18.70, 96.60, and 70.40% (Figure 1C), respectively. The optimal culture temperature was investigated, and the preferred temperature was 28°C according to the ester conversion ratio. The respective conversion ratios were 6.90, 24.50, 89.80, and 72.00% (Figure 1D). The rotation rate can affect the enzyme production by affecting the dissolved oxygen concentration of fermentation medium and the dispersion of fungal species (Feng et al., 2003). The preferred rotation rate for producing enzymes responsible for esterification was 250 r/min after an optimization of rotation rate. The conversion ratios of ethyl valerate, ethyl caproate, ethyl caprylate, and ethyl caprate were 7.87, 29.20, 94.80, and 85.20% (Figure 1E), respectively.

In this study, *A*. *niger* CGMCC 3.4309 showed a good performance on esterification by determining the conversion ratios of ethyl valerate, ethyl caproate, ethyl caprylate, and ethyl caprate. The conversion ratios of the four ethyl esters increased for 3.53, 5.97, 2.14, and 3.02 folds, respectively, after an optimization of cultural conditions (Figure 1F). Meanwhile, the concentrations of the reaction substrates were added according to the concentrations of such chemicals during the actual baijiu fermentation process (Xu et al., 2021b). The concentration of caprylic acid or capric acid each was 10 mM, and the conversion ratios already reached 44.30 and 28.20%, respectively, before optimization. With enough substrates, the conversion ratios of ethyl caprylate and ethyl caprate could be further improved under optimized conditions.

This was the first identification of an *A*. *niger* species for efficient fatty acid ethyl ester synthesis in an aqueous-phase system. So far, only a few studies reported the ester synthesis of fungal species from baijiu in the aqueous phase system, such as *M. purpureus* and *C. lusitaniae* (Fan et al., 2021; Xu et al., 2021b). It was not a frequently identified property of the microorganisms from baijiu. This served as the reason for the unstable ester synthesis during baijiu fermentation. Even the strains of the same species showed different performances for esterification such as the *M*. *purpureus* species as reported in our previous work (Xu C. et al., 2020). This indicated the genetic divergences between the strains of the same species. The strains with efficient esterification properties might harbor specific enzymes responsible for esterification. *A*. *niger* CGMCC 3.4309 could simultaneously synthesize ethyl valerate, ethyl caproate, ethyl caprylate, and ethyl caprate in aqueous phase. Thus, it was necessary to investigate the possible enzymes for esterification.

### Screening of the Possible Enzyme for Ester Synthesis in Aqueous-Phase System

A BGIseq-500 platform was used to assemble the transcriptome data and generated a total of 24,319 unigenes (Supplementary Table 1). The unigenes were annotated to the seven functional databases NR, NT, Swissprot, COG, KEGG, GO, and Pfam, respectively. Finally, 22,396 (92.09%) unigenes were annotated by the NR database, 23,223 (95.49%) by NT, 14,469 (59.50%) by Swissprot, 12,901 (53.05%) by COG, 15,484 (63.67%) by KEGG, 16,476 (67.75%) by GO, and 16,153 (66.42%) by Pfam. The genes with transcriptional divergences were analyzed (Supplementary Figure 1). A total of 23 genes were upregulated in transcription and encoded the proteins with the potential for esterification (Table 2 and Supplementary Table 2). Among them, 15 genes were cloned, including An408, An605, An923, An1097, An1486, An2017, An2076, An2794, An3040-3, An3131, An3196, An3559, An3936, An5004, and An5100 (Supplementary Figure 2a). SDS-PAGE indicated 11 of the enzymes were expressed, except An2017, An3196, An3559, and An5004 (Supplementary Figure 2b).

**TABLE 2:** Transcriptional level comparison of the genes from *Aspergillus niger* CGMCC 3.4309 under differentiated conditions.

Gene number (renumber)	Annotation	Annotation pathway		Transcription level (Group 1 vs. Group 2)^a^
		KEGG	GO	
CL163.Contig2 (An163-2)	Lipase	K16815	GO:0016042	3.21
CL163.Contig6 (An163-6)	Lipase	K16815	GO:0016042	2.69
Unigene286 (An286)	Lipase	K14788	GO:0016787	2.17
CL408 (An408)	Lipase	K16815	GO:0016787	2.33
CL605 (An605)	Pectinesterase	K01051	GO:0016829	4.36
CL923 (An923)	Carboxylesterase	NA	GO:0016787	2.39
CL1097 (An1097)	Esterase	K01050	GO:0016787	3.74
Unigene1486 (An1486)	Lipase	K01050	GO:0080030	2.32
CL1556.Contig4 (An1556-4)	Carboxylesterase	K02332	GO:0016787	4.92
CL2017 (An2017)	Triglyceride lipase	K13333	GO:0009395	3.76
CL2076 (An2076)	Acetylesterase/lipase	K01050	GO:0016787	3.04
CL2505 (An2505)	Triglyceride lipase	K01956	GO:0016787	2.43
Unigene2588 (An2588)	Esterase	K03927	GO:0016787	2.06
CL2763 (An2763)	Esterase	K14675	GO:0004806	2.32
Unigene2794 (An2794)	Lipase	K09252	GO:0016787	2.85
CL3040.Contig1 (An3040-1)	Lipase	K16815	GO:0004372	8.89
CL3040.Contig3 (An3040-3)	Lipase	K16815	GO:0004372	7.69
CL3131 (An3131)	Esterase	K17648	GO:0016787	2.27
CL3196 (An3196)	Lipase	K12389	GO:0016787	2.36
Unigene3559 (An3559)	Lipase	K02332	GO:0080030	2.40
Unigene3936 (An3936)	Carboxylesterase	K01049	GO:0080030	3.40
Unigene5004 (An5004)	Lipase	NA	GO:0016042	2.95
Unigene5100 (An5100)	Feruloyl esterase	NA	GO:0016787	2.15

*^a^Group 1 is the species cultured under the optimized conditions, and Group 2 is the species cultured under the initial conditions.*

**FIGURE 2:**
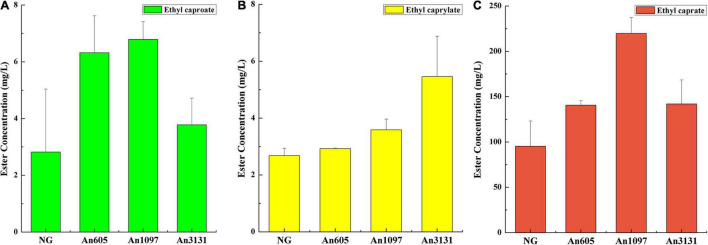
Esterification property investigation of the enzymes from *A. niger* CGMCC 3.4309. **(A)** Synthesis of ethyl caproate. **(B)** Synthesis of ethyl caprylate. **(C)** Synthesis of ethyl caprate. NG, negative control, the crude enzyme solution of *E. coli* BL21/pET-28a(+); An605, the crude enzyme solution of *E. coli* BL21/pET-An605; An1097, the crude enzyme solution of *E. coli* BL21/pET-An1097; An3131, the crude enzyme solution of *E. coli* BL21/pET-An3131.

Generally, enzymatic esterification preferred an organic-phase reaction system (Bollinger et al., 2020). Only some studies investigated an enzymatic synthesis of fatty acid esters in an aqueous-phase reaction system (Sun et al., 2012; Sun and Liu, 2015). In this study, some of the enzymes from *A*. *niger* CGMCC 3.4309 could synthesize fatty acid ethyl esters in an aqueous-phase reaction system. Esterification reaction showed that the enzyme encoded by An605 could synthesize ethyl caproate and ethyl caprate. The enzyme encoded by An1097 could synthesize ethyl caproate, ethyl caprylate, and ethyl caprate. The enzyme encoded by An3131 could synthesize ethyl caprylate and ethyl caprate (Figure 2). The results indicated that the esterification performance of *A*. *niger* CGMCC 3.4309 were the co-works of several enzymes (Figure 2).

### Enzymatic Property Investigation

**FIGURE 3:**
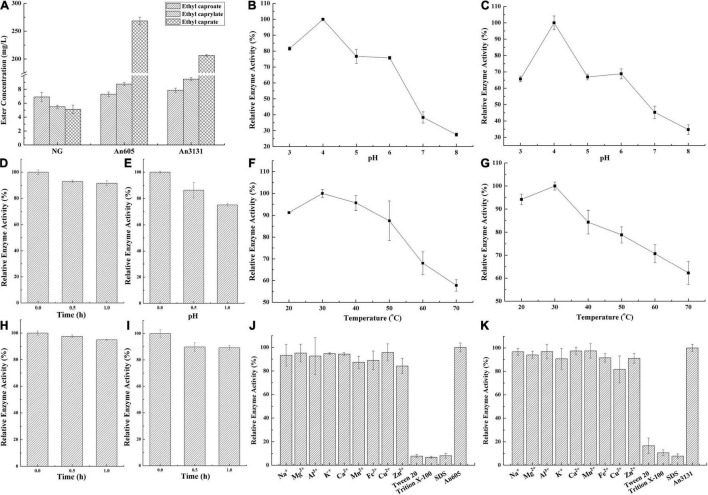
Property analysis of the enzymes numbered An605 and An3131 from *A. niger* CGMCC 3.4309. **(A)** Substrate preference. NG, negative control, the reaction mixture without enzyme solution. **(B)** Optimal pH of An605. **(C)** Optimal pH of An3131. **(D)** pH stability of An605. **(E)** pH stability of An3131. **(F)** Optimal temperature of An605. **(G)** Optimal temperature of An3131. **(H)** Temperature stability of An605. **(I)** Temperature stability of An3131. **(J)** Effects of metal ions and common surfactants on enzyme activity of An605. **(K)** Effects of metal ions and common surfactants on enzyme activity of An3131.

The substrate specificity of ester synthetic enzyme is determined by the activity of catalyzing the synthesis of esters with different chain length fatty acids as the substrates. In this study, An605 and An3131 were purified and the substrate specificity were investigated, while An1097 was failed to purify (Supplementary Figure 3). The substrate specificities of An605 and An3131 were the same, and both of them showed preference toward long-chain fatty acid capric acid (Figure 3A). The concentrations of ethyl caprate catalyzed by An605 and An3131 were 52.36-fold and 40.26-fold higher than that of the negative control, respectively.

The optimal pH and pH stability were investigated. The optimum pH values of An605 and An3131 were both 4.0 (Figures 3B,C). Acidic conditions were suitable for esterification, while in pH 8.0, the remaining enzymatic activities of An605 and An3131 were only 27.50 ± 1.08% and 34.77 ± 2.92%, respectively, when compared with the enzyme activities in pH 4.0 (Figures 3D,E). Compared with An605, An3131 showed a poor performance about pH stability (Figure 3E). After incubating An605 and An3131 in a reaction buffer at pH 4.0 for 1 h, the residual enzyme activity of An605 was 91.60 ± 4.44%, and An3131 was 75.10 ± 3.40%, indicating An605 was much stable at pH 4.0 compared with An3131 (Figures 3D,E). Temperature is also an important factor affecting enzyme activity. The optimal temperatures of An605 and An3131 were both 30°C (Figures 3F,G). When the temperature reached 70°C, the relative enzyme activities of An605 and An3131 dropped to 57.75% ± 2.66% and 62.27% ± 4.98%, respectively (Figures 3F,G). After incubating the pure enzyme solution at 30°C for 1 h, the residual enzyme activity of An605 was 95.00 ± 0.92% (Figure 3H), and that of An3131 was 87.80 ± 4.92% (Figure 3I), indicating that both of the enzymes showed good performance toward temperature stability.

Metal ions and surfactants could affect enzyme activity. In this study, Na^+^, Mg^2+^, Al^3+^, K^+^, Ca^2+^, Mn^2+^, Fe^2+^, Cu^2+^, Zn^2+^, Tween 20, Trition X-100, and SDS with a final concentration of 2 mM were added into the respective reaction mixtures to investigate their effects on enzyme activities. Results indicated that all the ions (Al^3+^, Mg^2+^, Cu^2+^, K^+^, Na^+^, Zn^2+^, Mn^2+^, Fe^2+^, and Ca^2+^) had little effect on the enzyme activity of An605 (Figure 3J). The metal ion Cu^2+^ showed a slight inhibitory effect on the enzyme activity of An3131 (Figure 3K). Surfactants greatly inhibited the enzyme activities of An605 and An3131. After being added with Tween 20, Triton X-100, or SDS, the residual enzyme activities of An605 and An3131 were all lower than 20% compared with the respective control (Figures 3J,K). Studies indicated that SDS could break the intramolecular and intermolecular hydrogen bonds of the enzyme, resulting in molecular unfolding, conformational change, and destruction of the enzyme (Zhang et al., 2018). Tween 20 and Triton X-100 are non-ionic surfactants, with a critical micelle concentration (CMC), that is, the concentration of aggregation to form micelles (Goswami, 2020). The CMC of Tween 20 was 0.06 mM, and the CMC of Triton X-100 was 0.2–0.9 mM (Goswami, 2020). The final concentration of the surfactant in the reaction system was 2.0 mM and was higher than their respective CMCs. The formation of micelles would affect the interaction of the enzyme with substrates, thus generating inhibitory effects.

### Homology Modeling and Molecular Docking

#### Homology Modeling and Molecular Docking of An605, An1097, and An3131

**FIGURE 4:**
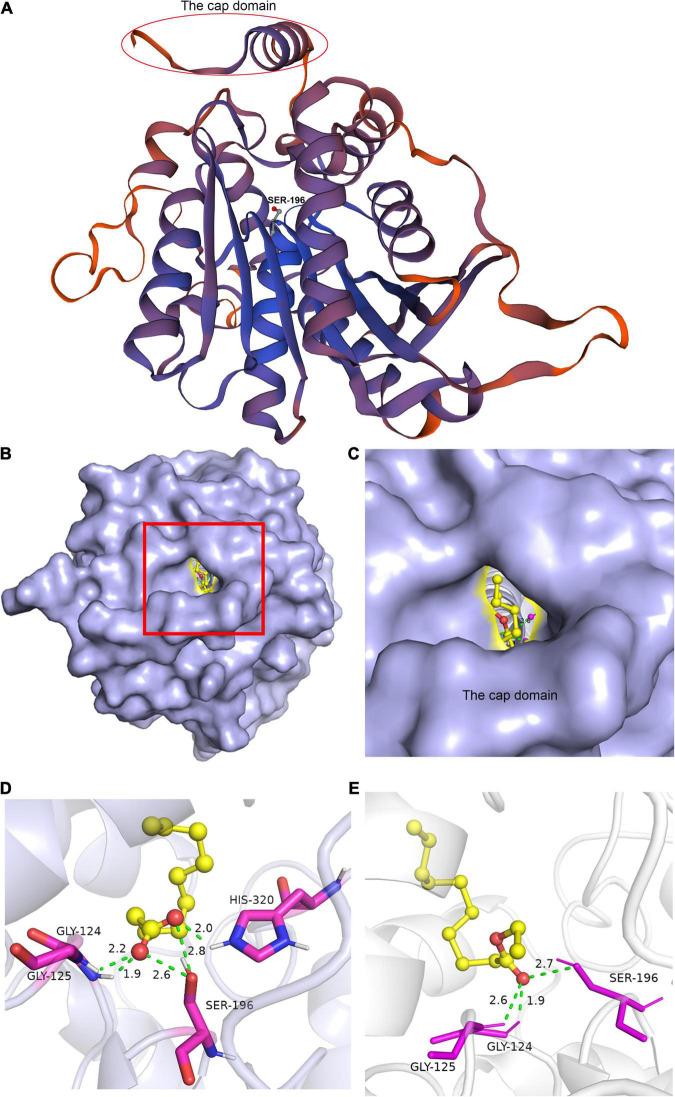
Homology modeling of An3131 and molecular docking of An3131 with the ligands. **(A)** Homology modeling of An3131. **(B)** Molecular docking of An3131 with ethyl caprate. **(C)** The catalytic active center of An3131. **(D)** The interactions of the amino acids with capric acid. **(E)** The interactions of the amino acids with ethyl caprate.

Homology modeling is a useful tool for investigating a protein structure. Combined with molecular docking, the possible enzyme–substrate interactions could be evaluated and provide useful information for the catalytic processes and for enabling interventions such as targeted mutagenesis (Tunyasuvunakool et al., 2021). Using An3131 as an example, the homology modeling was achieved using an alkaline esterase as the reference (PDB accession number 4YPV) (Pereira et al., 2017). The catalytic active sites were predicted by sequence alignment with the reference sequence of 4YPV (Supplementary Figure 4). An3131 contained a cap domain and the conserved sequence of GX_1_SX_2_G and was a candidate of the enzyme family of EC 3.1.1.1 (Figure 4A). The catalytic active center was constructed based on the spatial location of the catalytic active sites of the enzyme (Supplementary Figure 4). The molecular docking of An3131 with caproic acid, caprylic acid, capric acid, ethyl caproate, ethyl caprylate, and ethyl caprate were realized, respectively (Figure 4). Docking with capric acid indicated that the enzyme An3131 could interact with the ligand by hydrogen bonds through the residues Gly-124, Gly-125, Ser-196, and His-320 (Figure 4D). Ser-196 formed two hydrogen bonds with the two oxygen atoms (C=O, and C–O) of the carboxyl group of the substrate through the hydroxyl oxygen atom of Ser-196 (Figure 4D). All the docking results were summarized in Table 3. The docking of An3131-capric acid showed the lowest binding energy, indicating the highest affinity of An3131 to capric acid, and was in accordance with the enzyme property analysis (Figure 3A). The other two enzymes were also docked with the fatty acids and ethyl fatty acids (Table 3). An1097 was also one of the enzymes that belonged to EC 3.1.1.1 through sequence alignment and molecular docking (Supplementary Figure 5 and Table 3), while An605 was a candidate of the enzyme family EC 3.1.1.11 (pectinesterase) (Kent et al., 2016). The sequence alignment of An605 indicated that Gln-145, Gln-167, Asp-168, and Asp-189 might compose the catalytic center after comparing with the enzyme pectinesterase (PDB accession number 5C1E, identity 99.67%) (Supplementary Figure 5a; Kent et al., 2016). No cap domain was reported of this enzyme that might affect catalysis. The results indicated that the catalytic processes for esterification were differentiated from the enzymes from *A*. *niger* CGMCC 3.4309.

**TABLE 3:** The binding energies of the ligands with the receptor proteins An605, An1097, and An3131, and the amino acid residues formed hydrogen bonds with the distances.

Enzyme	Parameter	Caproic acid	Ethyl caproate	Caprylic acid	Ethyl caprylate	Capric acid	Ethyl caprate
An605	Binding energy (kcal/mol)	−4.80	−5.10	−5.50	−5.70	−5.80	−6.00
	Residue formed hydrogen bond (the distance, Å)	Gln145 (2.6) Gln167 (2.5)	Gln145 (2.6)	Asp189 (2.0) Gln167 (2.2) Gln145 (2.3)	Ser110 (2.6)	Asp189 (2.1) Lys311 (2.4) Tyr178 (2.2) Ser180 (2.4)	Leu116 (2.2)
An1097	Binding energy (kcal/mol)	−1.57	−3.75	−2.17	−4.14	−2.70	−4.11
	Residue formed hydrogen bond (the distance, Å)	Ser303 (3.3)	Ser219 (2.7) His456 (2.2)	Ser219 (2.6) Ser219 (3.3) Ser303 (2.7)	Ala413 (3.3)	Ser219 (2.7) Ser219 (3.1) Ser303 (2.8)	Ser219 (2.7) His456 (2.2)
An3131	Binding energy (kcal/mol)	−3.47	−4.25	−3.82	−4.53	−4.30	−4.78
	Residue formed hydrogen bond (the distance, Å)	Gly124 (1.9) Gly125 (2.2) Ser196 (2.6) Ser196 (2.9) His320 (2.1)	Gly124 (1.9) Gly125 (2.1) Ser196 (2.5) Ser196 (2.1) His320 (2.1)	Gly124 (1.9) Gly125 (2.1) Ser196 (2.6) Ser196 (2.8) His320 (2.0)	Gly124 (2.7) Ser196 (1.9) Ser196 (3.0) His320 (2.4) His320 (2.5)	Gly124 (1.9) Gly125 (2.2) Ser196 (2.6) Ser196 (2.8) His320 (2.0)	Gly124 (1.9) Gly125 (2.6) Ser196 (2.7)

#### Homology Modeling and Molecular Docking of the Unexpressed Enzymes

Due to the fact that only 11 of the 23 up-transcripted genes were heterologously expressed, the homology modeling and molecular docking of the left 12 enzymes not successfully expressed were carried out with the fatty acids and ethyl esters to further evaluate the esterification potential of these enzymes. Caproic acid, caprylic acid, capric acid, ethyl caproate, ethyl caprylate, and ethyl caprate were used as the ligands to dock with the enzymes, respectively. Six of the enzymes (An286, An1556-4, An2017, An2588, An3559, and An5004) built a 3D structure after homology modeling using the proteins in the PDB database as references. All the ligands could dock to the catalytic regions of the respective enzymes and with varied interactions and binding energies (Supplementary Table 3). It is generally recognized that more interactions between the enzyme and the chemical molecules and the lower binding energies mean the higher affinity between enzyme and the chemical molecules (Williams et al., 2004; Liao et al., 2017). This increases the probability of enzymatic reaction. The formation of hydrogen bonds between the enzyme and the fatty acids or ethyl fatty acids, the distances, and binding energies were summarized in Supplementary Table 3.

The results indicated that An2017 showed a substrate preference toward caproic acid and caprylic acid; An1556-4 and An5004 showed a substrate preference toward caprylic acid; and An286, An2588, and An3559 showed a substrate preference toward capric acid according to the binding energy evaluation (Supplementary Table 3). Sequence alignment showed that all these six proteins harbored the conserves GX_1_SX_2_G sequence, and the catalytic active site was serine (Supplementary Figure 5). The left six enzymes An163-2, An163-6, An2505, An2763, An3040-1, and An3196 showed a quite-low sequence identity with the reference enzymes with known structures (less than 10%). RoseTTAFold was used for the structure prediction of these enzymes (Baek et al., 2021). As less information about the reference sequences, the catalytic active sites of these proteins needed to be further verified to predict their catalytic properties (Supplementary Figure 6).

The catalytic triad of enzymes that belonged to EC 3.1.1.1 or EC 3.1.1.3 was generally composed of Ser-His-Asp/Glu (Rauwerdink and Kazlauskas, 2015). The catalytic active region is usually covered by a cap domain (Yu et al., 2014; Rauwerdink and Kazlauskas, 2015). The alter of the cap domain exposed the catalytic active center and allowed the substrates to enter into the catalytic active center for esterification (Yu et al., 2014). About 3–5 Å around the hydroxyl group of serine was a groove-like structure, and a pair of hydrogen atoms from the amino acids could be used as hydrogen donors. The structure was usually called “oxygen anion hole,” and was crucial to stabilize the transition complex formed during the catalytic process (Ferrer et al., 2015). The cavity between the oxygen anion hole and the key catalytic site serine was the substrate binding region.

**FIGURE 5:**
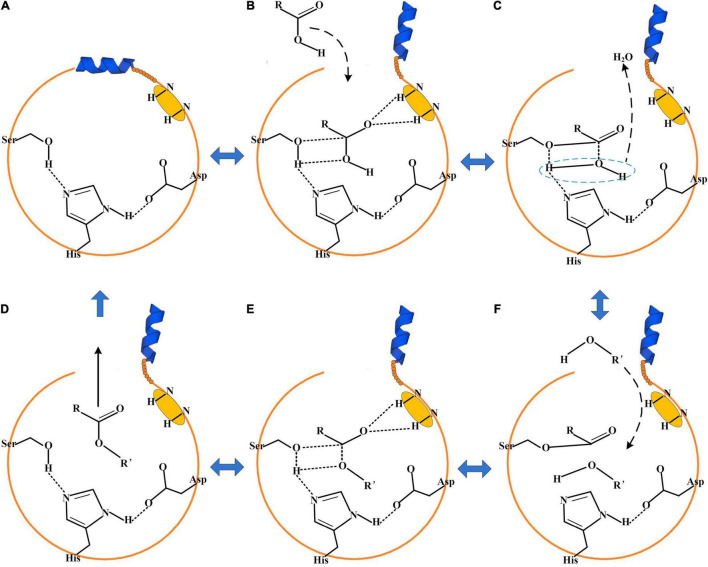
The proposed mechanism for enzymatic esterification. **(A)** The cap domain of the enzyme was closed. **(B)** When the cap domain altered, the substrate acid entered into the catalytic active center. **(C)** Tetrahedral transition state 1. **(D)** The substrate alcohol entered into the catalytic active center. **(E)** Tetrahedral transition state 2. **(F)** Esterification completed.

According to the above analysis and literatures about enzymes that belonged to esterase/lipase, the enzymatic esterification reaction was a reversible process (Ferrer et al., 2015; Rauwerdink and Kazlauskas, 2015). When the cap domain altered, the substrate molecules entered into the catalytic active center of the enzyme (Figures 5A,B). The side chain hydroxyl oxygen atom of serine nucleophilically attacked the carboxyl carbon atom of the substrate. The electron was transferred to the carboxyl oxygen atom of the substrate to generate an oxygen anion and formed a hydrogen bond with the hydrogen atom of the oxygen anion hole to maintain the stability of enzyme–substrate complex. A covalent bond was formed between the carbon atom, substrate, and oxygen atom of the serine residue, generating a tetrahedral transition state 1 (Figures 5B,C). Thereafter, a water molecule was removed to form acyl-serine (Figures 5C,D). The alcohol entered into the catalytic active center and attacked the carbonyl carbon atom of acyl-serine to form tetrahedral transition state 2 (Figure 5E). Finally, the carbon atom of tetrahedral transition state 2 formed a covalent bond with the alcohol oxygen atom, and the covalent bond between the side chain hydroxyl oxygen atom of serine and the carbon atom of substrate was broken to realize the esterification reaction (Figure 5). Meanwhile, not all the esterification enzymes from *A*. *niger* CGMCC 3.4309 harbored the catalytic active residual serine like An605, indicating that the esterification mechanisms of the enzymes followed different catalytic mechanisms and needed to be further revealed.

Previous studies focused on ester synthesis using enzymes from *A. niger*, while most of the studies indicated that the enzymes preferred an organic-phase reaction system for esterification (Table 4). In an aqueous-phase reaction system, these enzymes mainly showed the performance on ester bond hydrolysis (Bourne et al., 2004). The sequences of the reported enzymes were differentiated with the enzymes in this work (Supplementary Figure 6), and the substrate preference was also distinctive. Based on differential culture conditions, transcriptome sequencing, gene library construction, enzymatic properties determination, and bioinformatics analysis, this work identified enzymes for fatty acid ethyl ester synthesis in aqueous-phase systems for the first time. This provided a molecular basis for ester synthesis in an aqueous-phase system catalyzed by *A. niger* CGMCC 3.4309.

**TABLE 4:** Esterifying enzymes from *Aspergillus niger* and the characteristics.

Enzyme name	Strain	Accession number	Characteristics	References
Lipase	*A. niger* NCIM 1207	—	Esterification was carried out at 30°C in 25 mL stoppered flasks with 10 ml *n*-hexane containing fusel oil (1.9 mL), acetic acid (360 uL) and 1 g enzyme. the product concentration of isoamyl acetate was 50.0 ± 2.8 g/L in 96 h.	Mhetras et al., 2010
Lipase	*A. niger* F0215	An14g02170	The ethyl lactate, butyl butyrate, and ethyl caprylate flavor esters were produced by esterification of the corresponding acids with conversion efficiencies of 15.8%, 37.5%, and 24.7%, respectively, in a soybean oil-based solvent system.	Cong et al., 2019
Ferulic acid esterase	*A. niger*	—	The esterification reaction mixture contained 1% ferulic acid in the presence of 85% glycerol and 5% dimethyl sulfoxide at pH 4.0 acetate buffer and 50°C, and 81% of ferulic acid could be converted to 1-glyceryl ferulate.	Tsuchiyama et al., 2006
EstA	*A. niger*	AY456379	EstA could hydrolyze vinyl acetate with *k*_*cat*_ and *K*_*m*_ of 400 and 2.7, respectively; vinyl propionate with *k*_*cat*_ and *K*_*m*_ of 160 and 2.9, respectively; triacetin with *k*_*cat*_ and *K*_*m*_ of 265 and 15, respectively, at 25°C, pH 5.0 and 0.1 M NaCl buffer.	Bourne et al., 2004
Lipase	*A. niger*	—	AN0512Lip exhibited high tolerance for various polar organic solvents with log*P* < 0.8, and the highest lipase activity (476% of its original activity) was achieved after addition of 90% (v/v) isopropanol to the reaction mixture. AN0512Lip also displayed 3-regiospecificity and great affinity for the long-chain fatty ester.	Liu et al., 2015
Feruloyl esterase	*A. niger*	—	Suitable conditions for esterification of ferulic acid with diglycerol were 100 mg of ferulic acid in the presence of 1 g of diglycerol and 0.1 mL of 1 M phosphate buffer (pH 6.0) at 50°C under reduced pressure. Under these conditions, 168 mg of feruloyl diglycerols was obtained, corresponding to a 95% conversion rate of ferulic acid.	Kikugawa et al., 2012

In general, enzymatic ester synthesis in the organic-phase reaction system is more efficient than that in the aqueous-phase reaction system. However, for a fermented food manufacturing process, it is impossible to provide an organic-phase reaction condition. Therefore, it is of great significance to investigate the enzymatic synthesis of flavor esters under the aqueous-phase reaction system. This can not only clarify the synthetic mechanism of flavor esters by *A*. *niger* but also helps to realize the stable synthesis of flavor substances through rational regulation of the fermentation process by microbes or enzymes, so as to ensure the product quality.

This work mainly focused on the catalytic synthesis of short- and medium-chain fatty acid ethyl esters according to the flavor ethyl esters in baijiu. This did not mean that the catalytic substrate spectrum of the strain and its related enzymes were limited to the substrates used in this work. Other substrates such as medium- and long-chain alcohols and fatty acids could be used as the substrates to further reveal the esterification ability of the strain and related enzymes. These investigations will promote the application of *A*. *niger* CGMCC 3.4309 and its enzymes for flavor ester synthesis in food industry.

## Conclusion

A strain of *A. niger*-designated CGMCC 3.4309 was characterized to efficiently synthesize fatty acid ethyl esters in an aqueous-phase reaction system. The optimal culture conditions for producing esterifying enzymes included a culture temperature of 28°C and rotation rate of 250 r/min, with the culture medium composed of lactose as the carbon source and (NH_4_)_2_SO_4_ as the nitrogen source. The conversion ratios of ethyl valerate, ethyl caproate, ethyl caprylate, and ethyl caprate reached 7.87, 29.2, 94.8, and 85.2%, respectively. Compared with the initial cultural conditions, the conversion ratios of the above four esters were increased by 3.53, 5.97, 2.14, and 3.02 folds, respectively.

Based on transcriptome sequencing, gene library construction, enzymatic property analysis, homology modeling, and molecular docking, three enzymes with ester synthetic abilities were identified, and the ester synthetic potentials of other enzymes were evaluated. For example, the enzyme numbered An2017, An1556-4, and An5004 might have the ability to catalyze the synthesis of ethyl caprylate. The possible catalytic mechanism of esterification was proposed. These studies provided a technological combination for a comprehensive analysis of the ester synthetic enzymes in aqueous phase derived from microorganisms during the baijiu manufacturing process.

## NCBI Nucleotide Accession

The RNA sequencing data were submitted to NCBI Sequence Read Archive under accession numbers (SRX12524232), (SRR16244411), (SRX12524233), (SRR16244410), (SRX12524234), and (SRR16244409) for Group 1 samples and (SRX12524688), (SRR16244867), (SRX12524689), (SRR16244866), (SRX12524690), and (SRR16244865) for Group 2 samples.

## Data Availability Statement

The datasets presented in this study can be found in online repositories. The names of the repository/repositories and accession number(s) can be found in the article/Supplementary Material.

## Author Contributions

YX contributed to the methodology, investigation, data curation, writing–original draft, and funding acquisition. HH and MW contributed to the methodology and investigation. HL contributed to the data curation and writing–original draft. ML, CSZ, ZZ, CNZ, and WL contributed to the data curation. XL and BS contributed to the resources, writing–review and editing, funding acquisition, and supervision. All authors contributed to the article and approved the submitted version.

## Conflict of Interest

The authors declare that the research was conducted in the absence of any commercial or financial relationships that could be construed as a potential conflict of interest.

## Publisher’s Note

All claims expressed in this article are solely those of the authors and do not necessarily represent those of their affiliated organizations, or those of the publisher, the editors and the reviewers. Any product that may be evaluated in this article, or claim that may be made by its manufacturer, is not guaranteed or endorsed by the publisher.

## Footnotes

1
https://robetta.bakerlab.org/

2
http://ekhidna2.biocenter.helsinki.fi/dali/oldstyle.html
