# Supplementary material for: Characterization of an Aspergillus niger for Efficient Fatty Acid Ethyl Ester Synthesis in Aqueous Phase and the Molecular Mechanism
Source: Front Microbiol. 2022 Feb 21;12:820380. doi: 10.3389/fmicb.2021.820380 (PMC8899536; doi:10.3389/fmicb.2021.820380)
Supplement: Supplementary file 1 [file Data_Sheet_1.docx]

Supplementary information for

**Identification and characterization of an *Aspergillus niger* for efficient fatty acid ethyl ester synthesis in aqueous phase and the molecular mechanism**

Youqiang Xu^1,3,#,*^, Huiqin Huang^1,#^, Hongyun Lu^1,#^, Mengqin Wu^1,#^, Mengwei Lin^1^, Chunsheng Zhang^2^, Zhigang Zhao^2^, Weiwei Li^1^, Chengnan Zhang^1^, Xiuting Li^1,3,*^, Baoguo Sun^1,3,4^

^1^ Beijing Advanced Innovation Center for Food Nutrition and Human Health, Beijing Technology and Business University, Beijing 100048, China.

^2^ Chengde Qianlongzui Distillery Company, Hebei 067400, China.

^3^ Beijing Engineering and Technology Research Center of Food Additives, Beijing Technology and Business University, Beijing 100048, China.

^4^ Key Laboratory of Brewing Molecular Engineering of China Light Industry, Beijing Technology and Business University, Beijing 100048, China.

^#^ These authors contributed equally to this work.

* Correspondence:

X. Li, Beijing Technology & Business University. No. 33, Fucheng Road, Haidian District, Beijing 100048, China. E-mail: [lixt@btbu.edu.cn](mailto:lixt@btbu.edu.cn)

Y. Xu, Beijing Technology & Business University. No. 33, Fucheng Road, Haidian District, Beijing 100048, China. E-mail: xuyouqiang@btbu.edu.cn


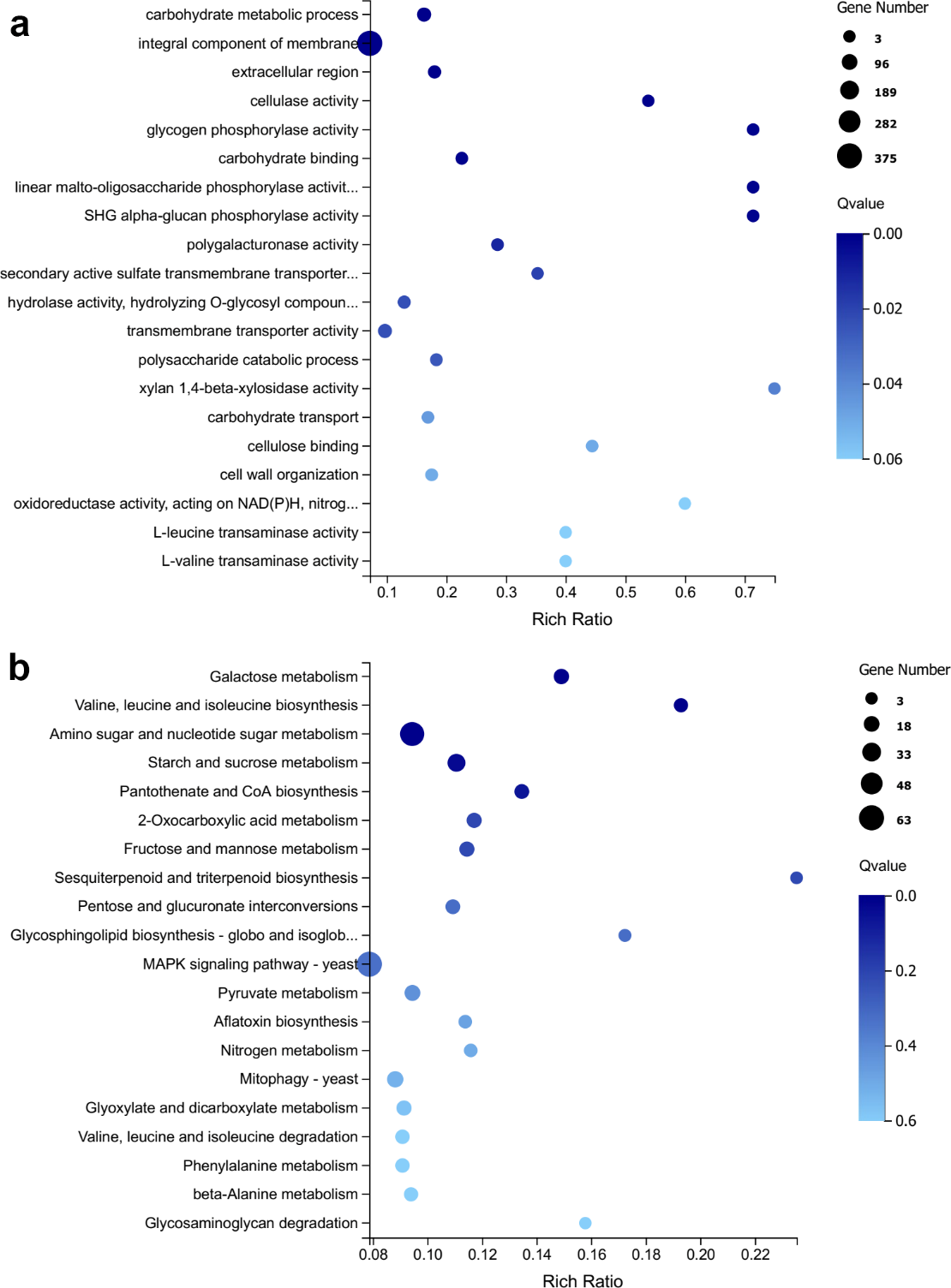


**Fig. S1.** GO (a) and KEGG (b) enrichment chart of genes with differential transcription levels.


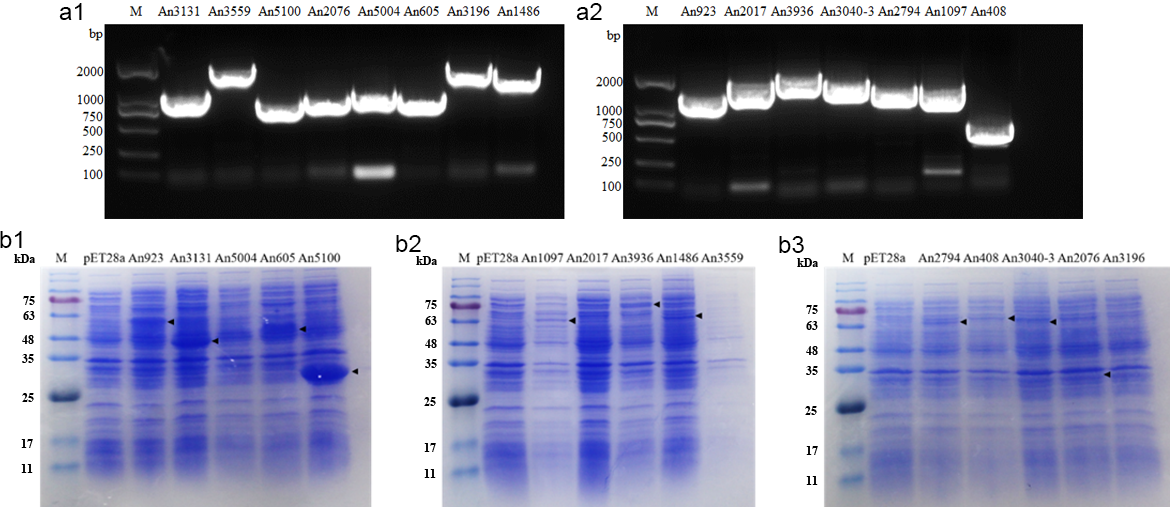


**Fig. S2**. PCR cloning of the 15 genes (a1, a2) and heterologous expressed in *E. coli* and verified by SDS-PAGE (b1, b2, b3).


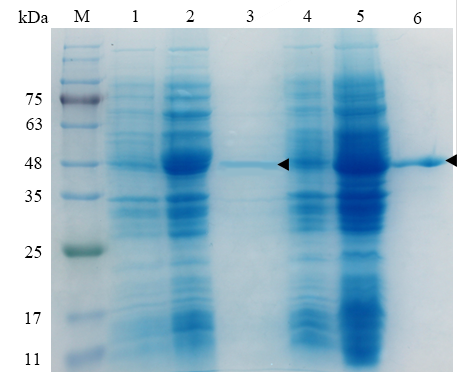


**Fig. S3**. SDS-PAGE electrophoresis of the purified enzyme An605 and An3131.

M, Marker; Lane 1, *E. coli* BL21/pET-28a(+); Lane 2, *E. coli* BL21/pET-An605; Lane 3, purified An605; Lane 4, *E. coli* BL21/pET-28a(+); Lane 5, *E. coli* BL21/pET-An3131; Lane 6, purified An3131.


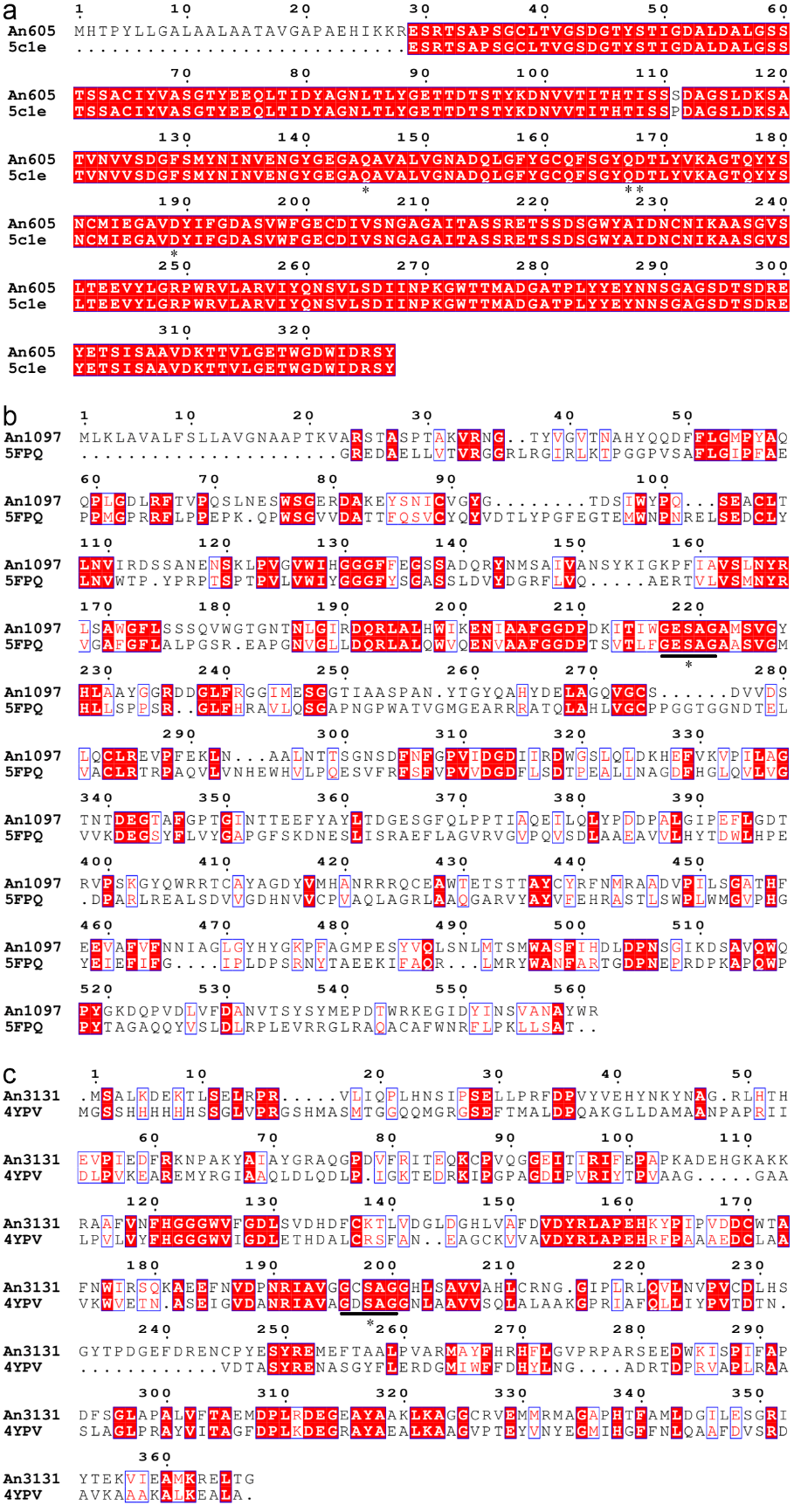


**Fig. S4**. Sequence alignment of the enzyme sequences in this work with the respective reference enzymes with three-dimensional structure.

The conserved regions were underlined, and the catalytic active sites were indicated by *.


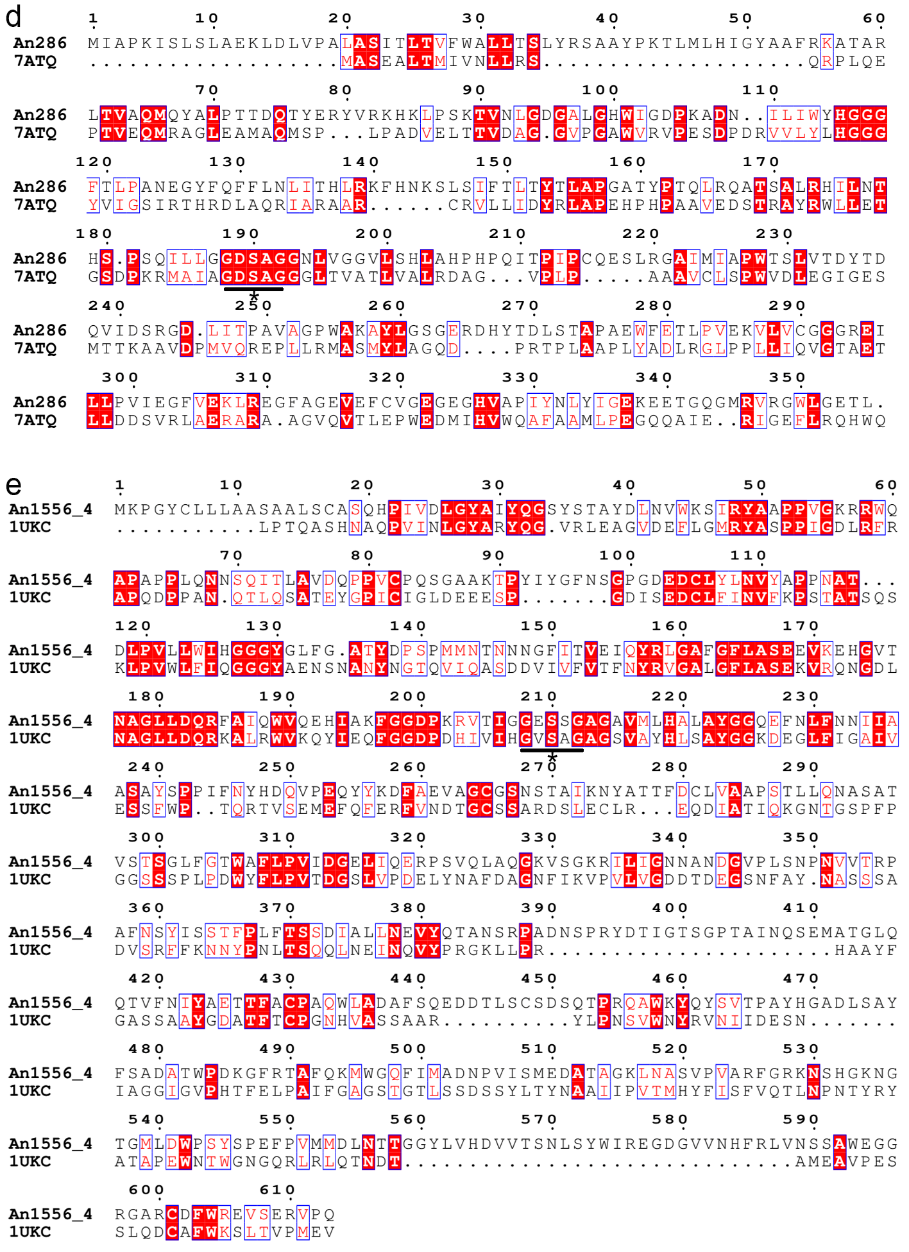


**Fig. S4**. Continued.


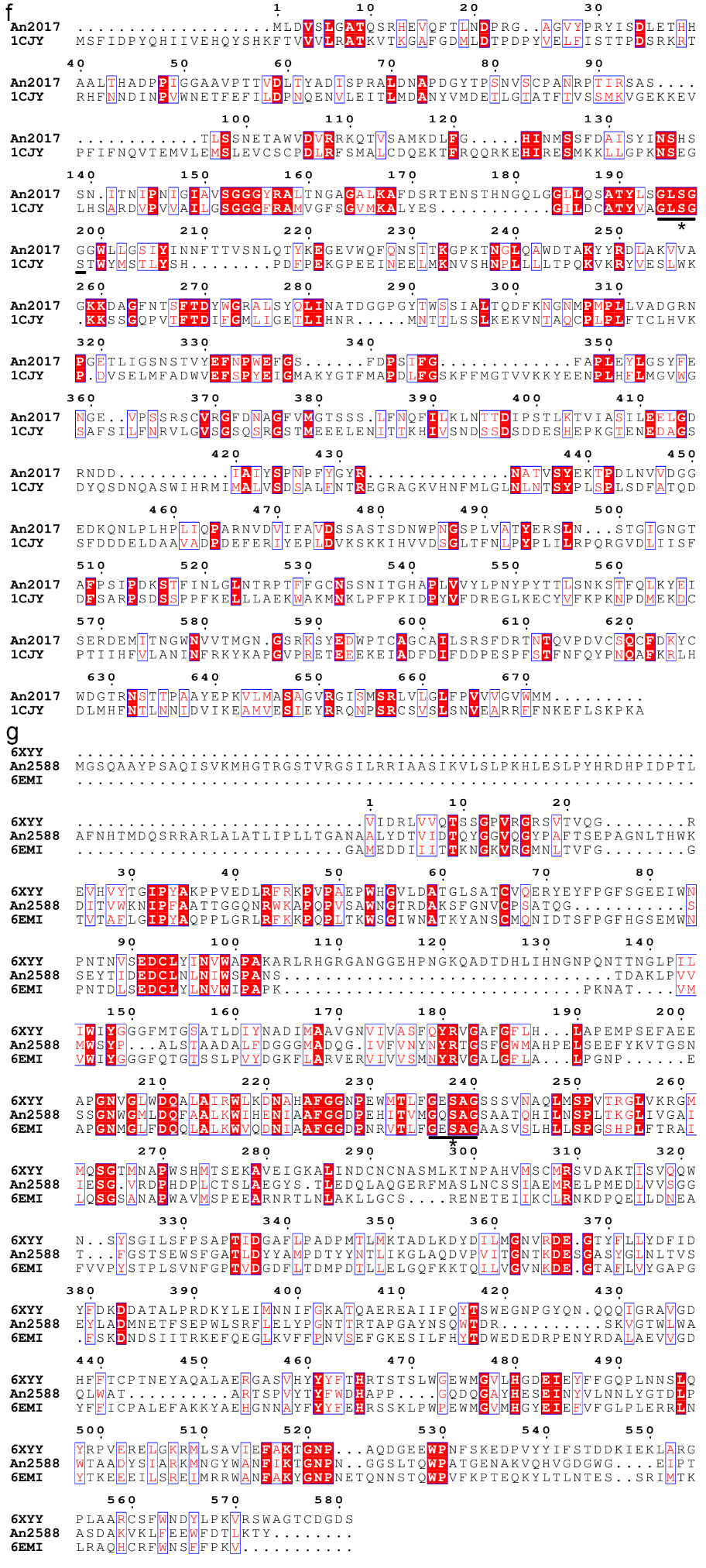


**Fig. S4**. Continued.


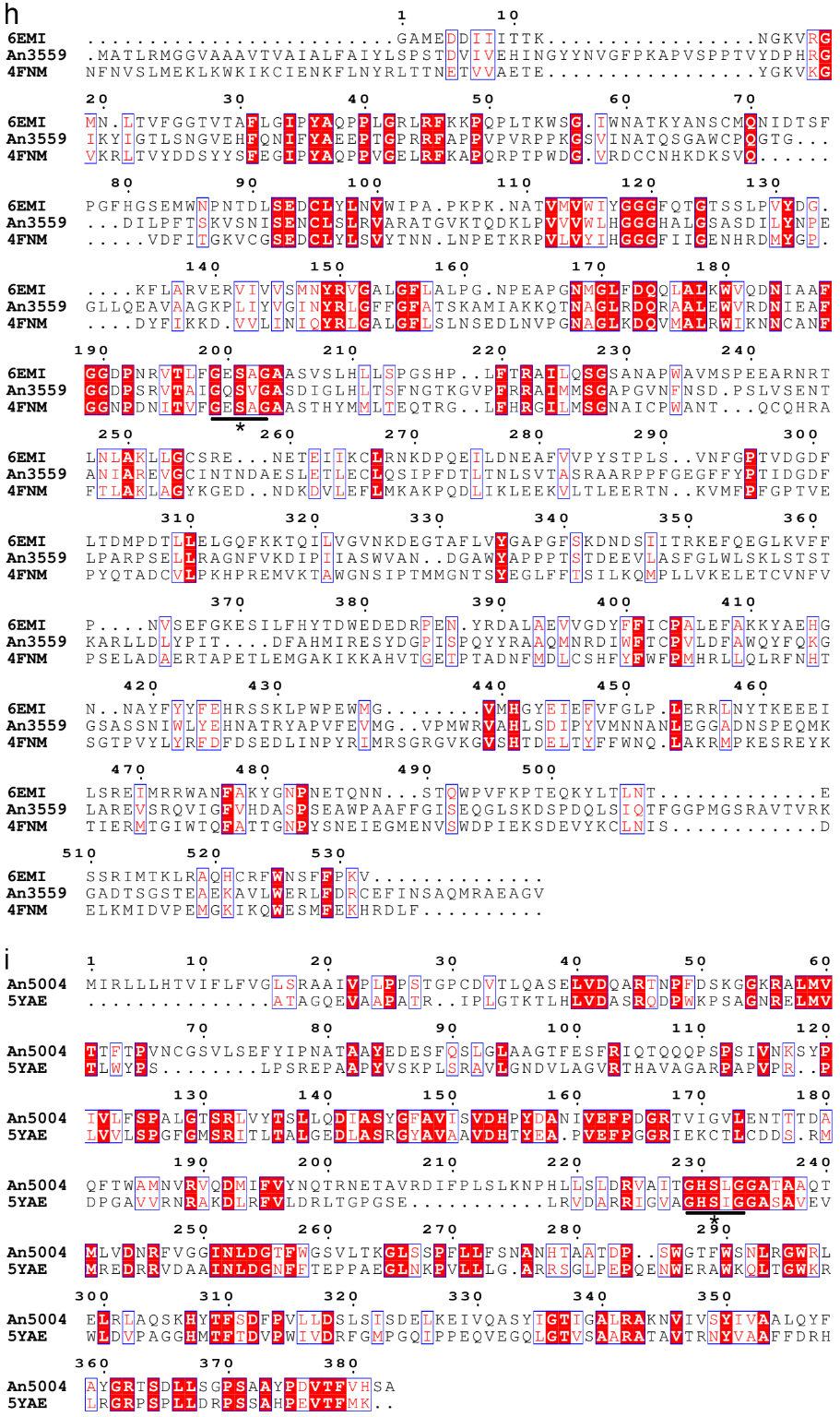


**Fig. S4**. Continued.


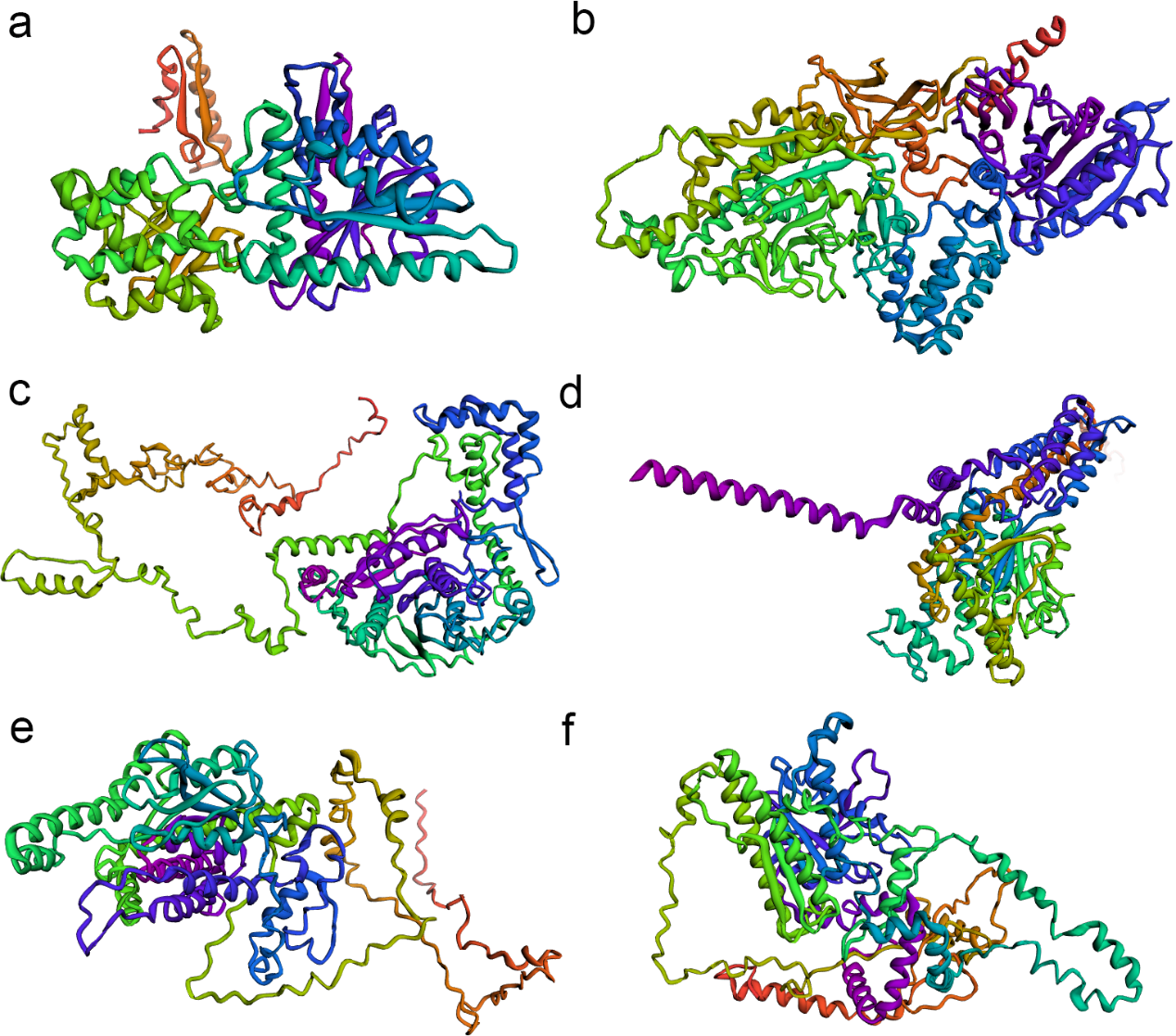


**Fig. S5.** Analysis of the three dimensional structure of the proteins by RoseTTAFold.

a, An163-2; b, An163-6; c, An2505; d, An2763; e, An3040-1; f, An3196.


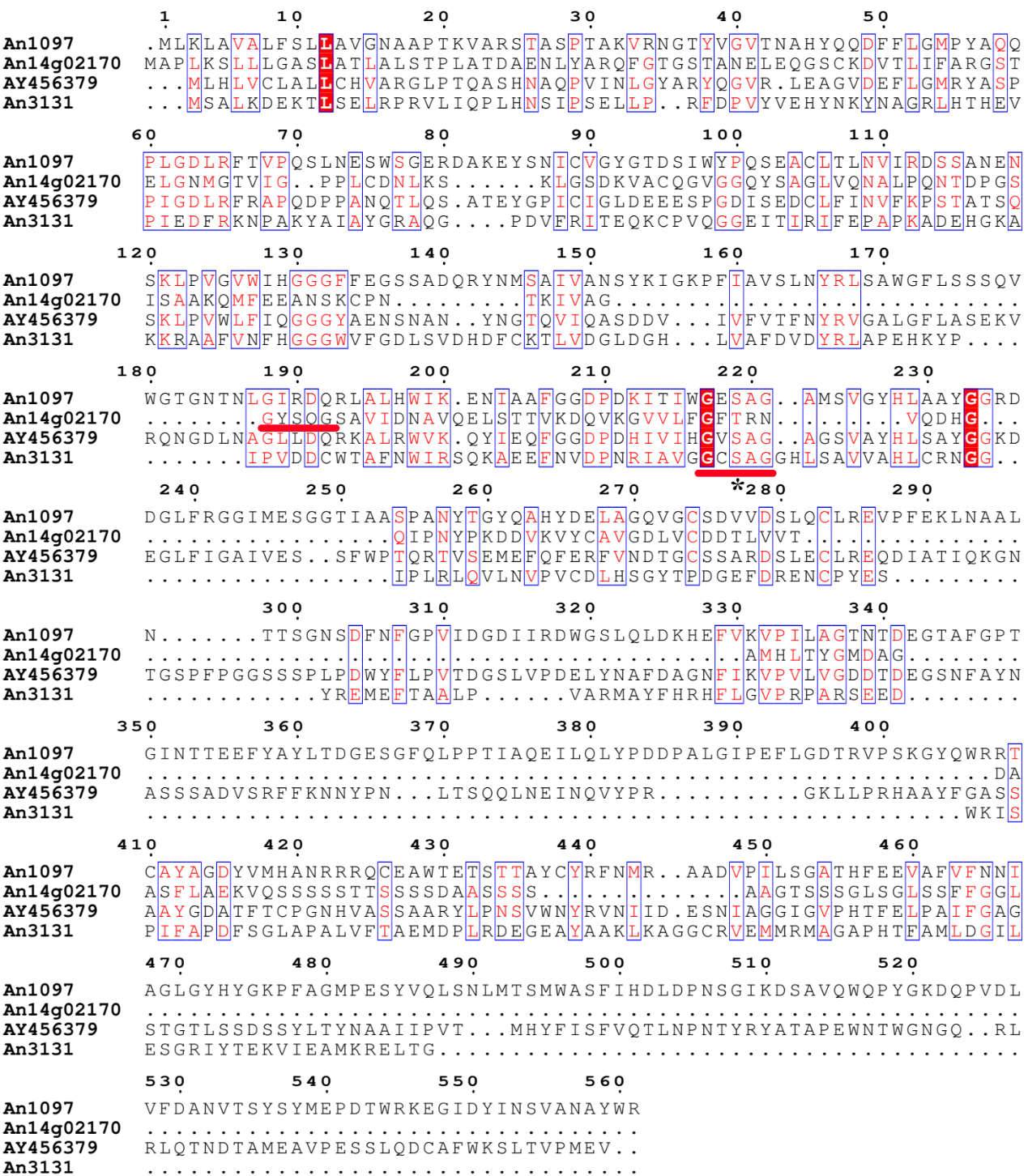


**Fig. S6.** Sequence alignment of An1097 and An3131 with the reported ester synthetic enzymes from *Aspergillus niger*.

The red line marked region was the conserved GX_1_SX_2_G region of the serine hydrolase, and S indicated the catalytic active amino acid serine (indicated by *).

**Table S1.** The unigenes of the transcriptome data.

| Group | Number of unigene | Total length | Mean length | N50 |
| --- | --- | --- | --- | --- |
| Group1.1 | 16,965 | 26,548,109 | 1,564 | 2,441 |
| Group1.2 | 17,211 | 27,647,433 | 1,606 | 2,488 |
| Group1.3 | 16,581 | 26,273,469 | 1,584 | 2,446 |
| Group2.1 | 19,288 | 32,884,411 | 1,704 | 2,590 |
| Group2.2 | 18,103 | 29,450,121 | 1,626 | 2,523 |
| Group2.3 | 20,220 | 35,216,184 | 1,741 | 2,684 |
| All-unigene | 24,319 | 48,254,046 | 1,984 | 3,005 |

^a^ Group 1 is the species cultured under the optimized conditions, and Group 2 is the species cultured under the initial conditions. .1, .2, .3 mean the replicates.

**Table S2.** The sequences of the 23 genes.

| **Gene ID** | **Sequence (5’-3’)** |
| --- | --- |
| An163-2 | ATGCGGCTCGCGCCCCAGACAGCCTGGCTGGCTCTCGTGGCGGAGGAGGCTGGTGCCTCCCTGCTGTATGATGCGACCCGGCCGAGGCAGCTGACGGCGGCAAGCACCCCTCGGCGCCCCTCACAGGCGGCCTTGGTTTGCTTCCTGGGCCGGAGCCGGAAAGACCGAGCGCTACGCTACCTGTTCCCGGACAACCCATCACGCAAAGTCGGAGGGTTTAACCTGCGGGTCGATCACCGCACGTGGCCCACAGAACGACCGATTCTCTTCGCTGATGGGGATCCGCTCCGGGACTGCCAGTTGCCCGACACCGAGCTGCTGGCCCGCGACGAGCCGATCCCCATTACCTGGAGCTCTGACCCGGCTCTGCCGCTGCAGGACGTCATTGTCGCAAGGTGCTTGCTTCCCTTTGCTCACGTCACATGCATCTTTGCTGCAGACCTCGGGGGACTGCCCAGTGTTCGCCGCCTCTTGAATCGATGGGCGGTAGCAGGCCGTGATGCCACGCGGCCGTCGCTGCAGACGCGTATCATTGTCCTGGTTGACGCGGACGAGGTGTCCGAGTCGGATGGGCAGGCCTTGTTCCAGCAACTGGACGGATCAGAGCTGTACGCGAGCGTGCATCTGCTCCCGGTGTCACGGGGCGACGTGCTCTCCGACGAGGCGAGGTATCGCCCGGTAAAGGAAGAGATTCTCAAAGCCCTGGACCGGGCGACCCGGGAGCGAGTGACGACACAGACATCTTTCTTGGCGTGTCATCTGGCCCGCTTTCTGGAGGGTGCTATCCGCCACACGGCTCAGGACCACCAGAAGCCGTTTGATCTGATCGCAGCGGGCCGCCCCCAGCCCCGACCAGCCGAGTGGGCTGCCTGCATCGGGGACTTTCTCGAGCAGACGCAGGCCATTGGCTCCGAGACGCAGGACCAGCTCCTCGCCTCGAGTCTCCTGCTGGACGCCTACCCGCCGGATACTCACGGATTCCACCCGCGCGATCTGTTCCGCCAACGCTACCGACAGCCCTGCCTGGACGCGCTGCGGGGGGTTGCATGTTCCGCTACAGCCACGGCTCGCGCCGACTCGATCGAGTCACGGCTGGTTGACGCCCATGCATGCCTGCTGGACCAGGACGTGATTCCGTTAGCGCTCCATGTGCAGCACCTGGCCGAATGGCGGGCGATTTTCGAACGTCTCCACAGCAACCGTACGTGTTTGGGTTGCCTTCTGTGCCGCCCGCAACATCCGCTGGCCTGTGGCCATGCCCTGTGTGACCGCTGCATCGAGCGTTACGGTCGACCCGCCCCGCGGAGAGAGTCGGCCTTTGTCGTCGAGACCTGCCCACTGTGTCAAGCTCCATGCGTCACATCGGTGGTTCTCTTGCCTCCCACCGCGGCTGTGCGTGCCCTCGCCGTGGATGGGGGCGGGGTTCGTGGCGTCATCCCCATCCGAATCTTGCTGGGCCTGCAACTCATCCTGGGGCCCAAATGCTCCCTTCTGGGGCTGATTGATGTCGCATTTGGCACCAGTGCCGGTGAGGATGGCAACACAACCTGTCCTTTTCCCTGA |
| An163-6 | ATGGCCTACATGCTGTACGCCTGGCTGGCTCTCGTGGCGGAGGAGGCTGGTGCCTCCCTGCTGTATGATGCGACCCGGCCGAGGCAGCTGACGGCGGCAAGCACCCCTCGGCGCCCCTCACAGGCGGCCTTGGTTTGCTTCCTGGGCCGGAGCCGGAAAGACCGAGCGTTACGCTACCTGTTCCCGGACAACCCATCACGCAAAGTCGGAGGGTTTAACCTGCGGGTCGATCACCGCACGTGGCCCACAGAACGACCGATTCTGTTCGCTGATGGGGATCCGCTCCGGGACTGCCAGTTGCCCGACACCGAGCTGCTGGCCCGCGACGAGCCGATCCCCATTACCTGGAGCTCCGACCCGGCTCTGCCGCTGCAGGACGTCATTGTCGCAAGATGCTTGCTTCCCTTCGCTCACGTCACATGCATCTTTGCTGCAGACCTCGGGGGACTTCCCAGTGTTCGCCGCCTCTTGAATCGATGGGCGGTAGCGGGCCGTGATGCCACGCGGCCGTCGCTGCAGACGCGTATCATTGTCCTGGTTGACGCGGACGAGGTGTCCGAGTCGGATGGGCAGGCCTTGTTCCAGCAACTGGACGGATCAGAGCTGTACGCGAGCGTGCATCTGCTCCCGGTGTCACGGGGCGACGTGCTCTCCGACGAGGCGAGGTATCGCCCAGTAAAGGAAGAGATTCTCAAAGCCCTGGACTGGGCGACCCGAGAGCGAGTGACGACGCAGACATCTTTCTTGGCGTGTCATCTGGCCCGCTTTCTGGAGGGTGCTATCCGCCACACGGCTCAGGACCACCAGAAGCCGTTTGATCTGATCGCAGCGGGCCGCCCCCAGCCCCGACCAGCCGAGTGGGCTGCCTGCATCGGGGACTTTCTCGAGCGGACGCAGGCCATTGGCTCCGAGACGCAGGACCAGCTCCTTGCCTCGAGTCTCCTGCTGGACGCCTACCCGCCGGATACACACGGATTCCACCCGCGCGATCTGTTCCGCCAACGCTACCGACAGCCCTGCCTGGACGCGCTGCGGGGGGTTGCATGTTCCGCTGCAGCCACGGCTCGCGCCGACTCGATCGAGTCACGGCTGGTTGACGCCCATGCATGCCTGCTGGACCAGGACGTGATTCCGTTAGCGCTCCATGTGCAGCACCTGACCGAATGGCGGGCGATTTTCGAACGTCTCCACAGCAACCGTACGTGTTTGGGTTGCCTTCTGTGCCGCCCGCAACATCCGCTGCCCTGCGGCCATGCCCTGTGTGACCGCTGCATCGAGCGTTACGGTCGACCCGCCCCGCGGAGAGAGTCGGCCTTTGTCGTCGAGACCTGCCCACTGTGTCAAGCTCCATGCGTCACATCGGTGGTTCTCTTGCCTCCCACCGCGGCTGTGCGTGCCCTCGCCGTGGATGGGGGCGGCGTTCGTGGCGTCATCCCCATCCGAATCTTGCTGGGCCTGCAACTCATCCTGGGGCCCAAATGCTCCCTTCCGGGGCTGATTGATGTCGCATTTGGCACCAGTGCCGGCGGGCTGATCATACTCGACATCTTTGCCAAGCACAGATCCGTGACAGACTGTCTGGAGGCTTTTCAGCGCCTGCTATTCCACTTCTTCCAGGGTCCACCATGCAGTCGTCTGTTCTCATGGCCACGCCGCATGATACGCAGTGCCCTGGGCCGAGGGCTATACGACGCGAACGAGCTCGAATCGCTTCTCCGCGCCCACTATAGCGATACACAGCGGCTTTTCAGCCCAGAGAGTAGGTCAGAGACCAAGGTCGCGGTCACCACCACCACTGAAGACGGTACGGCACTAGTGACTAATTATAAAGCCGCGACACTGCGGCCCCGCAATGTTGGCTACCGTAGTGTGCTTGCGAAGACGCCGGAGGAAGAGCCGTTGCTCTGGCAGAGTGCCCGCGCCACATCTGCCGTCCCCCTGCTTTTTCGCCCGCTGGTGCTGCCCGCGCTGGGCGCCTGTTGGGACGGGGGCCTGCGGCACAACAACCCAGTAGCGCTTTGTCGCCAGGAACTGCAATACATGTGGTCCTGGCAACCCCCACTTGGTATTCTCATTTCCATCGGCACGGGTACGCGAGGATCCCGCGCGAACAGTCCCGGTCCCGAACGGTCGTGCGCACCTGCCCCCCGTAGCCATTTCGTCTGGCCGGTGCTGCGTAGCTTCCTCGAGACCATGGACGGCAAGACATTGTGGGATCGCTTCCGCAACCAGGCGGCCGCGGCTGACCAGGACGCCTTGTCTCGACTGGACATCTGCCTTGCGGGACCGGAGCCCCGCCTTGATGCAGCCCATGCCATGGACGATCTCATCCGGCAAACCGTCGAACAGGGGGTGGGGAGACAGGGGCGCCAGTGCCTGCTGAGGCTCCTCGCGCAGAGTCTATTCTTCGAACTGGTCTCCGTTCCGGAGCGCGAGACGGGCACTCCGTCGTACTGGTGCGTCGGAACCATTCGGTGCCGGGTCTCCGGCGAGGTCTTTCTGAGCGCTATCCAACGAGTCGATGCATGCCAGATGGACTACGTCGTTGGCGGCAAGCCTCTGGGCATCAGCGTCACGGATGGGGCCACCTGCGCAAGGTGCGGGCGGTATTGCGTCCCTGTCCGGTTCCGCGTCCGCCGTCTGAGCGACACCGTTTCCCTATCACTCCGGCTGGACGACGGGCGACAGGTCCCCGTGGGCGGGTTCCCTAACCCGATGGATTGGTTTCTGCATCGGCAGGGATTGTCGCTTGCAGACGGCGTGACTGAACCTGGAGCCGCGTCCCGGGACGAGTGCGTGGCGTGTGAGCGCCGACTTGCGCTGCGGCGGGGTCTGCGGGTAACCCGACTGAAGGCCAGGCGGATGGTCCAGATTGCACACGCAATAGCTTGTGCAGCAGAAGAGGCCCAGGTTACTTACGAATAA |
| An286 | ATGATCGCTCCCAAAATCTCGCTCTCTTTGGCGGAGAAACTGGACTTGGTGCCAGCTCTCGCATCTATTACCTTGACAGTTTTCTGGGCCCTTCTGACCTCCCTTTATCGTTCTGCCGCATATCCCAAGACGTTGATGCTGCATATTGGCTATGCAGCATTTCGCAAAGCCACAGCCCGCTTGACAGTGGCCCAAATGCAATATGCTCTTCCAACCACTGATCAAACCTACGAACGTTACGTCCGGAAGCACAAGCTACCCTCCAAGACAGTAAACTTGGGCGACGGGGCTCTGGGACATTGGATCGGGGACCCGAAAGCGGACAATATCTTGATTTGGTATCACGGAGGCGGCTTCACCCTCCCCGCCAACGAAGGCTACTTCCAGTTCTTCCTGAACCTCATCACCCACCTGCGTAAATTCCACAATAAATCGCTCTCCATCTTTACGCTCACCTACACTCTCGCCCCGGGGGCCACTTACCCAACGCAACTGCGCCAAGCAACCTCCGCTCTCCGCCACATCCTTAACACGCACTCCCCTTCCCAAATTCTCCTTGGCGGGGATTCCGCAGGGGGGAACCTCGTCGGCGGAGTCCTCTCCCACCTCGCTCATCCCCACCCTCAGATCACCCCCATCCCCTGCCAAGAATCCCTCCGCGGAGCAATCATGATCGCACCCTGGACCAGTCTAGTCACGGACTACACCGACCAGGTGATCGATTCGCGCGGGGATTTAATTACCCCTGCTGTTGCGGGACCTTGGGCAAAGGCGTACCTTGGGTCCGGGGAACGGGACCATTATACGGATTTGTCCACTGCACCAGCGGAGTGGTTTGAGACCTTACCCGTGGAGAAGGTGTTGGTTTGTGGTGGCGGGAGGGAGATTCTGTTGCCTGTGATTGAGGGGTTCGTGGAGAAGTTGAGGGAGGGATTTGCAGGGGAGGTGGAGTTTTGTGTTGGGGAGGGAGAGGGACATGTGGCACCCATTTATAATTTGTATATTGGGGAGAAGGAGGAGACGGGGCAGGGGATGAGGGTGAGGGGGTGGTTGGGGGAGACTTTGTGA |
| An408 | ATGCTTGGCCGCTTGGAGATGGACATTGATCAGTGCATCAACGCATACAAAAGGCTGTCAAACCACGTATTTGCTCGAAAGCGACTTCTGCCAGTAGGAGGCAACTTGCGCCGACAATCGAAGTATGACGGTCGCAGGATTGAAACTGCCATGAAGGCGATTCTTCATGAGCTTGGCTACGAAGATGACATACTTCTAAGAGAACCGGCATCTTCCTGCAAAGTCTACGTTTGCGTGACCGATGACGGTTCAAAACGTAACCTAGCACCCTTGACAAGCTACCCGAGCAAATACTGCTCCAACGAGCTTTACAAGACCGCCAAAGTATGGCAGGCTGGTAGAGCCTGCTTCTCCACAGAGCAGCTCTTCGATCCCGTTCCTATTGGACCAAGCGGACGGCGATTTTACAATAGCAGCACAGACGCTAACAATCGCGTGCGAGAAGTCTGGATAGAAGCGAGAGGGATATGGCCATCCGGCTCATTGGATAACCAGATACGCTGCATGGTATCAATTGGCACGGGTGTGCCGTCGATCAAACGATCCGGCAAAGCGATGTTCGGCTTACTGAAGACCTCGCGCACTGATTCCATTGATACCGAGATGGACATGAACAAGTTCCTCCGAGAGCATACCGAACTAGACGACGAGAATCGTCTATTCCGATTTCGATGTCCCCAATGGCTTAGCGGATATTTCACTCGATGGGATTGA |
| An605 | ATGCATACTCCATATCTTCTGGGCGCCCTTGCCGCCCTGGCTGCTACCGCCGTCGGTGCTCCGGCAGAGCACATCAAGAAGCGAGAGAGCCGGACGAGTGCTCCCTCAGGGTGTCTGACGGTCGGATCGGATGGAACATATTCCACCATCGGCGACGCGCTTGACGCTCTAGGCTCATCCACTTCGTCCGCTTGCATTTACGTTGCCAGCGGCACGTATGAGGAGCAGCTGACCATTGACTACGCTGGCAACCTCACCTTGTACGGTGAGACTACCGACACCAGCACATACAAGGACAATGTGGTGACCATTACCCACACCATCTCGTCGTCGGATGCCGGCTCTCTCGACAAGAGCGCCACCGTCAACGTGGTCTCGGATGGGTTCAGCATGTACAACATCAACGTGGAGAATGGATATGGTGAGGGAGCACAGGCTGTAGCCCTTGTCGGAAACGCAGATCAACTGGGCTTCTATGGATGCCAATTCAGTGGTTATCAAGACACTCTCTATGTCAAGGCCGGTACCCAGTATTACTCCAACTGCATGATCGAGGGCGCGGTCGACTACATCTTCGGCGATGCGTCCGTGTGGTTCGGCGAATGTGACATTGTGTCTAACGGCGCCGGTGCCATCACGGCCTCATCGCGCGAAACCTCCTCTGACTCAGGCTGGTACGCTATCGACAACTGCAACATCAAGGCTGCTTCGGGAGTCTCTCTCACGGAGGAAGTCTACCTGGGCCGGCCGTGGCGCGTGCTGGCGCGAGTCATCTACCAGAACTCAGTGTTGTCGGACATCATCAACCCCAAGGGATGGACGACCATGGCAGACGGTGCGACGCCGCTGTATTATGAATACAACAACTCTGGTGCGGGATCGGACACATCTGATCGCGAATACGAGACCTCCATTTCTGCTGCGGTTGACAAGACCACAGTGCTGGGCGAGACTTGGGGAGACTGGATTGATCGGAGCTACTAA |
| An923 | ATGAAGAAGACGCTGCTCCTAGTGTTTATCCATGGCTTCAAGGGAGATGATGATACGTTTGGGGAGTTTCCAGGGCGCTTTTGTGCTCTAATTAGTCGCGCCCTCCCCGCTGTCCAGGTTGTCGCCACTGTTTACCCGAAGTATGAGACCCGAGGCGAACTCAAAGGATGCGTGAGTAATTTTCGGGAATGGCTTCAGAACAAAGTCATCGACCTGGAGGTCTCGAATCGAACGGCTTCACCGACAGTTGATCCTTCGGTACATGTTATTCTGCTCGGCCACTCTATGGGTGGGATAGTGGCTGCTGAGACCCTTCTTCTGCTTGCATCGGAACAGCCAATTCCTGCACCCTCTTCTGCACATTCTGCAACCGACACCCTAGTGGATTCTGAATCTGCGGTGGGGGCGCACTCTTTCATGTTCCCTCACATCCAGGGTGTTATCGCATTCGACACCCCCTTCCTAGGGATTGCCCCTGGTGTCGTATCGTATGGTGCAGAAGGCCATTACCGAAATGCCACTACGGCGTACACCGCGATCTCAGAAGTTGCTGGCTTGTTCGGCCTGGGTGGCGGTAACTCTTCGAACAAGGCAACTTCTACCGACCAGGAAAGAACTTCCAACAAGAGCCTACCTCCTTCGGGTCCTAGCTCGTCTTCAAGTGCAGATGCTGCCGCAACACCATCATGGCAACGATGGGGCAAATATGCCATGTTTGCAGGAGCTGCAGGAGCACTGGCTGCGGGCGGGGCGGCCGCAATGTATTCACAGCGAGATCGACTGACGGATGGCTGGGGCTGGGTATCATCCCACCTGTCATTTGTCGGCTCTCTAGCACGACCTTCTGAATTACGGAGGCGCCTTGCACTCCTTGCCGAAGTCCAAAGGGACCGAGGTATCGGCTGCACTAATTTCTATACGTGCTTGGGCAAAAATGCAAGTGATCTAGTGGAGAACAAGAACGCTGGGACTTCGTCGTTCAGCCAGAAGATTATACGCTCCAAAAATCGCACTTTCTGCTCGCTTCCCGCGGAAGTGGAAAGTCCGGCGTCTTCCCAGCAAGACATCCGATGGGCCAGGGCTGTGAACGATAAAGCTGCAGACGAAATCAAAGCACACATTAGCATGTTCTTGCCGAACGAGAATCCGGCCTTTAATTTACTTGTGTCCGATGCATGCAAAGTGCTGGTGGAGTCGGTTGACAAGGGCTGGTACGCCACGGCCACAGGGCCAATTGAAGAGGTGGACGGGAACATGCCTCGGTCCGACGATGACCGTAGCAAAGACCAGACTGATAGCGGATTTATGGACGGGGACGACGTTGTGGTCGTGGAGTGA |
| An1097 | ATGCTAAAGCTCGCTGTTGCTCTTTTTTCGTTACTTGCCGTGGGCAATGCAGCGCCAACCAAAGTGGCCCGTTCCACGGCCAGTCCTACGGCCAAGGTTCGCAACGGTACATATGTCGGAGTGACAAATGCGCATTACCAGCAAGATTTCTTTTTGGGAATGCCGTATGCCCAGCAGCCTTTAGGTGACTTGCGCTTCACGGTGCCTCAGTCCCTGAACGAAAGCTGGAGTGGCGAGCGCGACGCGAAGGAATATTCCAATATCTGTGTAGGATACGGTACCGACTCGATTTGGTACCCACAGTCCGAAGCTTGTCTAACCTTGAATGTCATCCGCGATTCTTCTGCAAATGAGAACTCGAAGCTCCCCGTGGGCGTCTGGATACATGGAGGTGGCTTCTTTGAGGGATCTAGTGCTGACCAGCGCTACAACATGTCCGCGATTGTTGCCAACTCCTATAAGATCGGAAAGCCGTTCATTGCTGTCAGCTTAAACTATCGCCTTTCGGCATGGGGCTTCTTGAGTTCCAGTCAAGTCTGGGGCACTGGCAATACCAATCTAGGTATCAGGGATCAAAGGTTAGCACTCCATTGGATCAAGGAGAATATCGCGGCATTCGGAGGAGACCCAGATAAGATCACTATCTGGGGCGAATCTGCCGGAGCGATGTCCGTGGGTTATCACCTTGCAGCATACGGCGGTAGGGACGATGGACTCTTCCGTGGAGGAATTATGGAGTCAGGAGGGACTATTGCAGCTAGTCCAGCCAACTATACCGGGTACCAAGCGCACTATGATGAGCTCGCGGGTCAAGTCGGTTGCTCCGACGTAGTAGATTCGTTGCAGTGCCTGCGCGAAGTTCCGTTCGAGAAATTGAACGCTGCTCTCAACACCACCAGTGGTAACTCGGATTTCAATTTCGGGCCCGTCATTGATGGAGATATAATCAGGGACTGGGGCAGCCTCCAGCTAGACAAGCATGAATTCGTCAAAGTCCCTATTCTTGCAGGTACCAATACCGACGAAGGGACAGCCTTTGGGCCCACAGGTATCAACACGACAGAGGAGTTCTATGCATATCTCACAGATGGCGAATCTGGATTCCAGCTACCCCCCACGATCGCCCAGGAAATCCTGCAGCTCTACCCTGATGATCCAGCACTGGGCATCCCCGAATTTCTCGGTGACACTAGAGTCCCGTCCAAAGGCTACCAATGGCGGCGCACCTGTGCATACGCAGGGGACTATGTAATGCATGCCAACCGTCGCCGACAATGTGAGGCGTGGACAGAGACCTCGACGACGGCGTACTGTTATCGATTCAATATGCGTGCGGCCGATGTCCCCATCCTGTCTGGCGCCACCCATTTTGAAGAAGTTGCTTTTGTATTCAACAACATTGCAGGACTCGGGTACCATTACGGAAAGCCGTTCGCAGGGATGCCCGAGTCCTACGTACAGCTAAGCAACTTGATGACCAGCATGTGGGCATCCTTCATCCACGATTTAGACCCTAATTCGGGCATCAAGGACTCAGCTGTACAGTGGCAACCGTACGGGAAGGATCAGCCGGTTGATCTAGTGTTTGATGCGAATGTCACGAGCTACAGCTACATGGAGCCAGACACGTGGCGGAAGGAGGGGATCGACTATATCAATTCCGTGGCCAACGCGTACTGGCGATAA |
| An1486 | ATGCATTTTTCTCATTCTTCTCTTCTTCTCGGGGCAATATGCGCATGGGGCCTTGCCAGCCCTTTGCCCTCAAGTACAAGTCCTACGGTGCATCTCCCTGATTCCAATGTCTGGTTCAAGGGAACGGCAGGCAACTTGACCGAATCCTTCCTCAATATCCGCTTTGGTGAAGACACGAGCGGGAGCAACCGCTTTGCTCCTCCGAAACCATACAACTATCCCTCCGGCTCCGTGGTGAATGCCACCCAGTCCGGCGCAGCTTGTCCTCAGCAAAAGGACCCTATTCCTGACTTTCCCATCTTCGACAATGTCACTTCCATCTCTGAGGACTGTCTGACGCTGCGCGTTGATCGGCCACTCAACACGTCTTCCTCGGACCGTCTACCAGTCCTCGTCTGGATCTACGGCGGCGGCGACACCATCGGCCAAATCTATGACTCTGCCTATGATCCAAGCCTTTTGGTCTACGGAGCTGCCCAGAAAGATGTTCCCGTTATCTACGTGGCTATGAACTACCGTCTCGGTATCTTCGGATTTGCCTCATCCCCCGCCTTGAACGATTCCGACTCCCTGAATGCCGGTCTGCTGGACCAGCGCTTAGCCCTATGGTGGATCCAAGACAATATTGCAGCCTTCGGCGGTGATCCAGACCAGGTCACCATCTTCGGCGAAAGTGATGGTGCAACAAGCGTCGGACTTCATATCACTGCCTATGGCGGCAAAGAGTCCAAAGTGCCCTTCTATCGCGCAATTATGGAATCAGGCGGCGCCACTGCCGACTCCGGAACGGCCAGCAACAAGTCAGGCGAGCACACAGCTACCCTAACCAGCCTGGTCAACTGCACTGCGTCCTCCAGCCAGGAGGAGCTCGAGTGTCTTCGTCGTCTCCCCATGAAGACCCTCCTCAACACAGCGTACGCCTACGAGCTCACTCTCAACTCCATGGGCGGCATGGGCGTTTTCATCCCCGCAGCCCCCTCACCCTTCATCCCCGATGCTCCGTCGAAACTCCTCCGAACTGGCCAATTCTCCCGCAACATCGACCTCATCTCCGGTTGGTGCGAGACTGATGGCTCATTCTTCACTACCACAACTCTGAACTCCTCATCCGACATCGCCGCCTTCCTCCAGGACAGCTACCCCAACCTCTCCAACGCCTCTATCGCCAAAGCCCTGGACCTCTACCCCGTGTCAGAATTCTCCCCGGACCCATCCGGAAACATCTCCGCGCAGTACTTCCGTGCTAGCCAGATGCACCGTGACCTCGACTTTACTTGTCCCAGTCTCTTGACTGTCCAGACGAACGTCAAGTACGCCACTGACAAATCCAAGGTTACTAACTACCTCTACGCACTCAACCAAACGGCCTTTGCAGAGGTCTTTGAGTACTTGGGCATGACGTACTACGGTGTTCCGCACTTTAGTGATATTATGTATCTTTTTAACTCTGTTACGAACGGCTTGTTCAAGGATGTATCGACAGCGTCGGATCGGAAGCTAGCGTCTGAGATGGGTGCCAGCTGGGCTTCGTTTGCCTACTATGGTAATCCAAGCCGTGCTAGGGGTAGTGTTAAGGGATGGGAGCAAGCGTATGCGGCGAATGCTAGTTCCACGACGCCTGAGTTGCAGGTCTTGGGTGGGCCGAAGATGAGGTTCACGGGTAGTGGGAAGGGAGCTGCGTATGATGAGGATTTGGTGAAGAGGTGCGCGTTTTGGAATGATCCGGTTGTGGCGAGTGAGTTGGGGGTGTAG |
| An1556-4 | ATGAAGCCCGGGTATTGCTTGCTGCTCGCTGCCAGCGCGGCACTCTCCTGCGCCAGCCAGCACCCCATCGTTGATCTGGGGTACGCGATCTATCAGGGCTCTTACAGTACCGCTTACGACTTGAATGTGTGGAAGAGTATCCGGTACGCAGCTCCCCCAGTGGGTAAACGGCGTTGGCAGGCGCCTGCACCTCCATTGCAGAACAACAGCCAGATCACATTAGCCGTCGACCAGCCCCCCGTCTGTCCTCAATCCGGGGCGGCAAAGACACCGTACATCTACGGCTTTAACTCGGGGCCTGGGGATGAAGACTGTCTGTACCTGAATGTCTATGCCCCCCCGAATGCGACGGACCTGCCCGTCCTGCTATGGATCCACGGTGGTGGCTATGGGCTCTTCGGTGCCACCTATGACCCCAGCCCAATGATGAACACCAACAACAACGGGTTCATCACCGTGGAAATCCAGTACCGTCTGGGAGCATTCGGTTTCCTCGCCTCCGAGGAGGTCAAGGAGCACGGTGTGACGAACGCTGGTCTTCTGGACCAACGCTTTGCCATCCAATGGGTACAGGAGCACATTGCCAAATTCGGTGGTGATCCTAAGCGTGTGACCATTGGCGGCGAGTCCTCAGGCGCCGGAGCAGTCATGCTGCATGCGCTGGCATATGGGGGTCAGGAGTTCAACCTCTTCAATAACATCATCGCTGCGAGCGCCTACTCACCCCCAATCTTCAACTACCACGACCAGGTACCAGAGCAGTACTACAAAGACTTTGCTGAAGTGGCCGGCTGCGGATCGAACTCCACTGCCATCAAGAACTACGCAACCACCTTTGACTGCCTGGTAGCAGCTCCCAGCACTCTCCTGCAGAATGCCAGCGCCACCGTCTCCACGTCCGGACTATTCGGCACATGGGCTTTCCTGCCGGTCATCGACGGCGAGCTGATCCAAGAACGGCCCTCTGTGCAGCTCGCACAAGGCAAAGTGAGCGGCAAACGCATTCTCATCGGCAACAACGCCAACGACGGCGTCCCCCTAAGCAACCCCAACGTAGTCACCCGCCCGGCATTCAACTCATACATCTCGTCCACCTTCCCCCTCTTCACCTCATCCGACATCGCGCTCCTCAACGAAGTCTACCAAACCGCCAACAGCCGACCCGCCGATAACAGCCCCCGCTACGACACCATCGGCACTAGCGGTCCCACGGCCATCAACCAATCCGAGATGGCAACAGGCCTCCAACAAACGGTATTCAACATCTACGCCGAAACGACCTTCGCGTGTCCCGCGCAATGGCTCGCCGACGCCTTCAGCCAAGAAGACGACACCCTTTCCTGCTCCGATTCCCAAACTCCTCGTCAAGCCTGGAAATACCAATACTCGGTAACACCCGCGTACCACGGCGCCGACCTCTCCGCGTACTTCAGCGCGGACGCCACCTGGCCCGATAAGGGATTCAGAACGGCTTTCCAGAAGATGTGGGGCCAGTTCATTATGGCTGATAATCCTGTGATTTCGATGGAAGATGCCACGGCTGGGAAATTGAATGCGTCGGTTCCTGTTGCGAGGTTTGGTCGTAAGAATAGTCATGGTAAGAACGGGACGGGGATGCTTGATTGGCCGAGTTATTCGCCCGAATTCCCGGTTATGATGGATTTGAACACCACGGGGGGGTACTTGGTTCATGATGTGGTGACGTCGAACTTGTCATATTGGATTCGGGAGGGAGATGGGGTTGTGAACCATTTTCGGTTGGTGAATTCGTCCGCTTGGGAAGGGGGAAGAGGGGCGAGGTGTGATTTCTGGAGGGAGGTTTCGGAGAGGGTACCTCAATAG |
| An2017 | ATGCTAGACGTGTCACTTGGTGCAACCCAAAGCAGACATGAAGTTCAATTCACTCTTAACGACCCTCGCGGCGCTGGGGTATATCCAAGGTATATTTCCGATCTTGAAACTCATCATGCAGCACTAACTCATGCTGACCCGCCCATAGGAGGCGCCGCGGTTCCTACAACCGTCGACCTCACATATGCAGACATATCACCTCGCGCACTGGATAATGCCCCTGATGGTTATACCCCGAGCAATGTATCCTGTCCTGCAAACAGACCGACGATTCGCAGCGCGTCAACCCTGTCATCGAACGAGACGGCATGGGTGGACGTCCGGCGTAAGCAGACTGTCTCAGCGATGAAAGACCTTTTCGGCCATATCAACATGAGCTCATTTGACGCTATTTCGTACATCAACAGCCATTCATCAAATATCACCAACATACCCAACATCGGTATTGCCGTGTCCGGCGGTGGCTACAGAGCCCTGACCAACGGCGCGGGAGCACTCAAGGCATTCGACAGTCGAACGGAAAACTCAACCCATAATGGACAGCTCGGTGGTCTTCTGCAGTCAGCCACATACCTGTCCGGTCTCTCCGGAGGTGGCTGGCTCCTGGGCTCAATCTACATCAACAACTTCACCACCGTCTCCAATCTGCAAACCTACAAAGAGGGCGAAGTCTGGCAGTTCCAGAATTCAATCACGAAAGGCCCAAAGACCAACGGCTTGCAAGCCTGGGATACAGCCAAGTACTACCGCGATCTGGCCAAGGTGGTCGCTGGCAAGAAGGACGCGGGCTTTAACACTTCCTTCACGGACTACTGGGGTCGCGCACTCTCCTACCAGCTGATTAACGCGACCGACGGAGGCCCAGGCTACACCTGGTCATCGATCGCTTTAACCCAGGACTTCAAGAACGGAAACATGCCCATGCCGCTCCTTGTCGCCGACGGCCGCAACCCAGGCGAGACCCTAATCGGCAGCAACTCGACCGTGTATGAGTTCAACCCCTGGGAATTCGGCAGTTTTGATCCGTCCATCTTCGGCTTCGCTCCCCTCGAATACCTCGGATCCTACTTTGAGAACGGCGAAGTCCCATCCAGCCGATCCTGCGTCCGCGGCTTCGATAACGCAGGCTTCGTCATGGGAACCTCCTCCAGTCTCTTCAACCAATTCATCCTGAAGCTCAACACCACCGACATCCCATCAACCCTCAAAACGGTCATCGCCAGCATCCTAGAAGAACTAGGCGACCGCAACGACGACATCGCCATCTACTCTCCCAACCCCTTCTACGGGTACCGCAACGCGACAGTTTCATACGAAAAGACCCCGGACCTGAACGTCGTCGACGGTGGCGAAGACAAACAGAACCTCCCCCTCCATCCTCTCATCCAACCCGCCCGCAACGTGGACGTCATCTTCGCCGTCGACTCCTCAGCCAGTACCTCGGACAACTGGCCCAACGGAAGTCCTCTCGTCGCGACTTACGAACGTAGTCTCAACTCAACCGGTATCGGAAACGGCACCGCGTTCCCTAGCATCCCGGACAAGAGCACCTTCATTAACCTGGGCTTGAACACCCGTCCGACTTTCTTCGGCTGCAATAGTTCCAATATCACAGGCCATGCACCCCTGGTTGTCTACCTCCCCAACTACCCCTACACAACCCTCTCCAACAAGTCGACCTTCCAGCTCAAGTACGAGATCTCGGAGCGTGATGAGATGATCACCAATGGCTGGAACGTGGTTACTATGGGTAATGGATCAAGGAAGTCTTACGAGGATTGGCCGACTTGTGCGGGCTGCGCTATTCTGAGTCGCTCGTTTGATCGGACTAATACCCAGGTGCCGGATGTGTGCTCGCAGTGTTTTGACAAGTATTGCTGGGATGGAACGAGGAATAGTACGACGCCGGCGGCGTATGAGCCGAAGGTATTGATGGCTAGTGCGGGTGTGAGGGGTATTTCGATGTCGAGGTTGGTTTTGGGTCTCTTTCCGGTGGTGGTTGGGGTTTGGATGATGTGA |
| An2076 | ATGACCGCGTATGGCGGCCGAGCGGAGGGACTCTTTGCCGGTGCGGTGCTGGAGTCTCCATTCTGGCCCACACTGCGCACGGTTGCCGAGATGGAATTTCAGTACACACGGTTTGTACATCGCGTCGGCTGCTCGGATGCATCGTCTGCCTTGGCGTGTCTTCGGTCTGCGGACCTGGCAGCGATACAGAAGGGCAACGTGGTGGACACATTTCCAGGCGCCGCCGTGGATCCGCCCCCGCTGTGGTATTTTCTGCCCGTCATTGACGGTGCGGTGGTGCAAGACCAGTTGTCTCGGCTCTTCGATCAGGGCAAGACCGTGAAAATTCCCGTGCTCGTTGGTGACGACACCAACGAGGGGTCGACCTTTGCTTATAACGCCAGCGACGCGTCTGACATGTCGCGGTTTCTCAACGCCAACTACCCAGGGCTGTCATCTGCCAGCCTGGCCGCCATCGGTGATGCATATCCTCGCATGCGACCCGTGGCGGATCATGCAGCCTATTTCCCGTCCGCGTCTGCCGCCTATGGTGACGCTGCCTTTACGTGCCCGGGCAATCGCATCGCAGCGTCCATGGCAGATCACCTCCCGTCCGGCAGGGTTTGGAGCTACCGATACAACGTGCGAGCCCCCCAGCTGGTCGATGAAGGCCTGGGTGTGCCGCATATCTTCGAGCTGAGCGCCATTTTCGGAGTCGGCTTCGCCGGTAATAGCGAGATCACGAGCTACAACGGGATCAACGCGAATGCGGTCGCCACCGTGATGGACTATTGGATCAGTTTCGTGAAGGCACTGGATCCGAACCCTCGCAGGCGTAGCCAAGCACCGCGCTGGGAAGCGTGGAAGCCCGGCCTGGGGCAACGGCTCAAAATACAGACAAACACTACCGCGATGGAGCCAATCCCGCCGCAGCAAGCGGATCGATGCTCACTGTGGAGCGCACTGGCGCCGGAAATGGAGATTTAG |
| An2505 | ATGAAGGATGATTACAACGAGCGGAAGCAGACGAGTCGCGATCATCCGAGCCAGAAGCGGATCATGCTGTATATCCACGGTGGGGCGTACTTTTTTGCTAGTCTGGATACCCATCGCTATCAGATGCAGCGACACGCTAGGAAACTCAAGGGTCGGGTTTTCGCACCTAAATATCGTCTGGCGCCGCAGTTTCCGTTCCCATGCAGTCTACAAGACTCGCTGGCCGCATACCTGTACCTGTTGGAGGATCATGATCCGAAAGAGATTATTTTCGCGGGAGATTCCGCCGGTGGAGGAATGGTGCTCTCAATGCTTGTCATTTGTCGCGATCAAGGACTGCCCTTGCCTGCGGGTGCTATTCTAATTTCTCCCTGGGTGGACCTCACGCATTCATTCCCCAGCATTGTCGCGGACAACCCGGGCGATTATATTCCTCCCTATGGCTTCAGGCATAAACCCTCCGCTGCATGGCCGCCGCCAAATGCGGACGAGCTGCGTGAGCTCAAGAAAGGCCTCAAAAAACACCCAGCCAGCCAGAGTGAGGCTCTTAAGGCCATCCCGAATGACAGCGAGGCCGAAGATACAGCAGCGAAGGGGTATTCGAGCCGTAGGAACAGCGCGGCCGATGCAAAACGTGCCTGCGATATCGAACACGAGACTGTCAAGGTGGTACTTGATGGGGAAACAGTGGAGGTCAAGGACCAGATTCACATGTACACGACCAACGAACTCTTGTCCCATCCTCTTGTTTCTCCTGTCTTTCAGCCGTCATTGGGGGGTCTACCTCCCCTGCTGATTCAAAGCGGAGGCGGCGAAATGCTACGAGACGAGCAGTTCTACATTGCACATAAGGCCGCCAACCCGACGGCATACCCGCCCAGTGACAAGTTCCTCGATGAGCATGATCCATCACGGGAGATCCTCCGCAAATACCCCGGTACCCATGTGCAGCTTCAAGTCTGGGACGACCTCTGCCACGTTGCTCCCACTCTCAGCTTCACGCGTCCAGCGAAGTATATGTACCGGGCGATCGCTCAATTTGGGGCCTGGGCCCTGGCATGTGCGCAGAAAGAGGAGGTGGAAATCGAAGATCAGCGGTCCGGGTCATCCAGCGACTCTGAAAACCTAGGAGACGCTACTAATGGCACAGCCAACGGCACTGCAGTCGCATCGGTTGGAAAAGCAGGCGATCCATTACCGGCCTTCAAAGATCGCATGATCCGTCAACGAGTCGACAAACGCGGTAATATCTACCCGCTTGATCCTCCATCCGCTTGCCCTGTTCTCCAGATTCCCAGCTCCGAAGTCGGTACTTTCAATCCCAAGCTTGTTCGGGGATGGCTGGCAGCCAAGGAGGAGTGGGACGAGAAATTCGCGAAGGAAAAGCTCCGGGTTCAGCGACAGCGGATCAAGGAACTGGCCTACGGGCTCCAAGCGTTCAAGGACGAGTTGCCTCCCCCCAGTTCACTCGCTGCGAGACGGCATGCCCCTGGCGTCTTGCCCTCCAAGACCGGCCGCAAGAGTTACCCCATGACCATGTGGAGCAGACTGGCCAGCAAGCAGGATGAAAGATCTATCGCGAAAGAACAGCTGAAAGGAGGCGAGGTCAAGCGAAAGAGTGTCGATGCCGGGCGCGCCGGAGCGTCAATGGACGAGGCCAACAGATCGAAAGCCGAGAGTTTACGCCCGTCCTCCCAACCGGCCACCCTTGCCTCGCATCCTACAAATATCGTCATTGTTCCAACCCGGTCAGAGAAAGGGAGCGTAGCCTCTCACAAGACGTCAACCGACGCAGTTGATCAGCTGGGAACCTCTCCCCCGCAGGGTACAGCTCTCAGTAAGTCCAGCAGCCGACTCATGTTACTACCCGAATACGAGGGGAAAACGCTCCTCGACGAGAACGCCAGCACAAGGACGCTTTTCCGTGAAGCCGGCGCAATCCCCACATCAGATGGCGCATGGTCACGGCTGCGCCAACGTCCATTGTCCCATGCCGGTTCCGGTACTATCCGCAGCGAATTTACCTCCGAGCTCCCTGAAGATAGCAGCACGATCGGCGATGAGAAGTCCCTTGCGGTAACCAACACAGGAGTCGACGAAGCCAGTACCCGTGCTGTGCTGCGCGCCGGTGGCGTGGTGGGACTGATGAGCGACGATGGCGACTCAGCGTATCGCTCGTTTGATGCGCAGTCGACAACGGATGCGGGGGATGCTGAATCTGCACCTGGAGGTGTCCATGGTGATTCTGGAGGGGTGAATGGCAGTGCTGAAGTTGACAAGCACGCGCGTCCGACCATGCCGGATCGCGAGTTCTTCAAGACAGCCGAGGAGTATGTGCCTTATTGA |
| An2588 | ATGGGATCGCAGGCCGCGTATCCCTCGGCGCAAATATCCGTGAAGATGCATGGCACCCGTGGGAGCACCGTTAGGGGGTCCATTCTCCGACGCATTGCGGCATCTATTAAAGTGCTGTCTCTCCCCAAGCATCTCGAATCATTACCTTACCATCGTGACCATCCCATTGATCCCACATTAGCGTTCAACCACACGATGGATCAGTCACGCCGTGCGAGACTCGCTCTCGCCACGCTTATTCCTCTCCTGACCGGGGCCAATGCAGCGCTCTATGACACTGTCATCGACACGCAGTATGGTGGTGTTCAAGGCTACCCAGCCTTCACCAGCGAGCCGGCCGGTAACCTAACCCATTGGAAAGACATTACCGTCTGGAAGAACATCCCCTTTGCCGCCACCACCGGGGGTCAAAACCGCTGGAAGGCACCCCAGCCCGTCAGCGCTTGGAACGGCACCCGTGATGCGAAGTCTTTCGGAAACGTATGCCCGTCGGCCACCCAGGGCAGCTCCGAATATACCATCGACGAAGACTGCTTGAACCTCAACATATGGAGTCCCGCCAACTCGACGGACGCAAAGCTCCCGGTTGTCATGTGGAGCTATCCCGCTCTGTCGACGGCTGCGGATGCCCTCTTCGATGGTGGCGGCATGGCTGACCAGGGTATCGTTTTTGTGAACTACAACTACCGCACAGGCTCATTCGGCTGGATGGCGCACCCGGAGTTGTCGGAGGAGTTCTACAAGGTGACCGGATCCAACAGCTCCGGCAACTGGGGCATGCTCGACCAATTTGCAGCGCTCAAGTGGATCCATGAGAACATCGCCGCGTTTGGCGGTGATCCCGAACACATCACGGTTATGGGCCAGTCTGCTGGGTCGGCTGCCACCCAGCACATCCTCAACAGCCCATTGACCAAGGGCCTGATCGTCGGCGCCATCATCGAAAGTGGAGTTCGGGATCCGCACGATCCCCTCTGCACCAGTCTGGCGGAAGGCTACAGCACATTGGAGGACCAGCTGGCCCAGGGAGAACGCTTCATGGCCAGCTTGAACTGCAGCTCCATCGCCGAGATGCGCGAACTGCCCATGGAGGATCTCGTCGTGTCGGGCGGAACATTCGGCAGCACCAGCGAATGGAGCTTCGGGGCCACTTTGGACTACTACGCAATGCCGGACACCTACTACAACACGCTCATCAAGGGTCTGGCGCAGGACGTGCCTGTCATTACGGGCAACACCAAGGACGAAAGTGGGGCCAGCTACGGCCTGAACCTCACCGTGTCCGAGTACCTGGCAGACATGAACGAGACGTTCAGCGAGCCATGGCTCTCTCGCTTTCTGGAGCTGTATCCCGGAAACACCACACGAACCGCCCCGGGAGCCTACAACTCCCAGTGGACCGACCGGTCCAAAGTGGGCACTTGGCTGTGGGCACAGCTCTGGGCGACCGCGAGGACCAGTCCTGTGTATACCTACTTCTGGGACCACGCTCCCCCAGGCCAGGACCAGGGCGCGTACCACGAATCCGAGATTAACTATGTTCTGAACAACCTGTACGGCACGGACCTGCCATGGACGGCGGCCGACTACTCCATCGCACGGAAGATGAACGGATACTGGGCCAACTTCATCAAGACGGGCAATCCCAATGGAGGATCCCTGACCCAGTGGCCTGCCACGGGAGAGAACGCGAAGGTGCAGCATGTGGGTGATGGATGGGGAGAGATTCCGACCGCATCTGATGCCAAGGTGAAGCTGTTTGAGGAGTGGTTCGACACACTGAAGACTTACTAA |
| An2763 | ATGGGGTCCTGGACGACCATCTGCGGACTATTCGTCGCTGTCCTAAACATGATACTCGACGTTGCCTTCTTCTGGAAAGAACGATTACTGTCTTGGTGGAGATCCAAGTCTCCTCGGAAGCGACTGCAACATGTTCTGAGGACAGCGTCCACCTTTGAGGAATGGGAAGAGGCTGCTTTCGAGCTTGACGAGCTCTTGAGCATGGACCTCTGGCGTCAGAATCCCACAAGCCGGCATTACGACTACCGACTGATCCTGGGTCGATTGGAGGCTCTTATGAGCGCTCGCGAGGATGAGGATATCCTGACCTTAGCGAACCTGCTTCGCTCCGGTTTGCTTCGGAATCTGGGCAACATTACCTCTCCCAAGATGTTTTTGCATGCCTACGCCGGCACCAAGCTACTCATAGATGACTATATTACGCAAGTTGCCCTCTCGATTCAACAGGTCACGGCTCTTCAAACTGCCCCGATTCACGACAGCGGGTTCTCGTCGCAAGCAAAGCTTGAACTTTTGCACGATACTCGGCAAGCCTTTGGGAGAACAACCCTACTGTTACAGGGCGGTTCAATGTTTGGACTCTGCCACCTCGGGGTAGTGAAGGCTCTCCATTTGCGTGGCCTCCTGCCCAGGATCATCACCGGGACAGGGATGGGGGCGCTCATTGCAGCATTGGTGGGTATCCATAGCGAAGATGAGCTGCTCACCTTTCTTGACAGCGGTGCCATCGATCTGAGCGCCTTCGATCGTAGACGACGGATGAAAAGGCCTGAAGAAGAGAGTTGGTGGTTGCCTTCTAATCTCGGGGACAGTTGGCTGGGGACACTATTGCGGCGAGTCAATCGATTCATTCAGAAAGGCTACTTCCTTGATGCAGAGGTGCTAGAGGAATGCGTTCGCGCCAATCTCGGCGACCTCACTTTCGAAGAAGCATATGCCCGGTCCAAGCGCATACTCAACATCACTGTTGCCACATCAAATAGAAACGCTACCCCGAACTTACTGAACTACCTGACTGCACCGAATGTGTTGATCTGGTCCGCTGCTGTAGCTTCCAATGCATCCAACAACTCACTTTACCAGCCAGTCACAATATTCTGTAAAGACGAAACAGGCTCGATTGTACCTTGGCCGCATTCTCGGGACGCAACCTTTTATCAATCCTGGCGTCAAGGTCACTACAAAGAGGAAGAATCACCGCTAGCCCGGATCGCCGAGCTATTCAATGTCAACCATTTCATCGTGTCGCAGGCCCGACCATACCTAATCCCTTTCTTACGCTCGGACTCGAACCTACTGGACCGTCGCCCAACCGATCAATGGAGCGTCACTCAAGCTCTAATGCGTCTCTTTACTGCCGAGCTCCACCACCGCCTCGGACAACTAGACTACATGGGCCTCCTTCCTGCCACTCTTGCACGGCTCCTCATCGAGGAGACCATCCCTGGCCCCCATCTTACCCTCGTCCCCGACGTCTCCCTCCTGGACCTCCCCAAGCTCTTCCAGAACCCAAGCAAAGACCGTCTAGCATACTGGTCTCTCAAAGGAGAGCGAGGCGTCTGGCCCGCCGTCAGCGCATTAAAAGTCCGCTGCGTCATCGAGATTGAGATAGACAAGGGCTACCAGGTTGTGCGTCGACGACAACCATCCGAAAATGCCCCATCCACGCCGCGTCGCATGCCCAACGAGGGACTCCCGCGTCGCCGTAGAGGCTATAGCATTGATTACGGTAGAGATATGCTAAATTTCTCTGGAAGTTTGGATTGTCAGGACGATTCGAATTATTAG |
| An2794 | ATGAAGTCAACTACACCTCTCCTCGCCGCTGCTGGCGTGGCTGCGGCCAAGAACTGCAGCGTTGACACCATTGCTAAATACCTTCCCAAGAATGCAACCGTCTTTTACGCCAATCATTATGAAAAGGGATATAACTTCACCCCTCCCATTGAATACAACTATGGCCTGACCTCCGATCCTATGGGCATCAGCGCCTATGAACTTCCTCTTGCTGGCTGTGTTGCCCAGGCCAACATCTCCCTGCCCAACAACACCCAGCACAGCGTTGGCTTAGTGCTACCCGATGAGTGGAACGGCAGATTCATGGCCGTCGGCAACGGTGAATTCGCCGGGTCTGTCGGCTGGTCTTCCATCATCAACACCATGTGGTACGGCTTTGCCAGTGTGTCAACCGACACCGGTCACGAGGGCAACAACGGAAGCTTCGGATACCACAACGAAGCTGCCCTCACCAACTGGGGCTACCGCGCCCTCCACGATGCCGTGGTCAACGGAAAGAAGGTTACCGAGGGCTACTACGAAAAGGACATCTCGTACAGCTACTACCGGGGTTGCTCCGCCGGTGGTAAGCAGGGCTTCAAAGAAGTCGAAATGTTCCCTGACGACTTCGACGGTGTGGTTGCCGGCGCACCGGCCTGGTGGACTTCTCACCAGCAGCTGTGGAACGTCCTCACTGCCATCTGGAACCTCCCCGAAACCGCAGACTACCACGTCAGCGATGCTCAAATGACCGCTGTCCAGGACGAGATCCTGAAGCAGTGCGATCCCCAGGACGGCCTGAAAGACAACATCCTGCAGAACCCCTTCGGATGTGTCTTCGACCCCGTCCCCGTGATGTGCAACGCCACCTCCTCCAACGACACCTGCGTGACCCCAGCCCAGCTCAAGACCGTGAACAAGCTCTTCAACCCCTGGTACGAAGCCAACGACACTCTGATCTTCCCGGGATACACCCTCGGCACTGAAGTCGGCGCCCCCAGCCTGGACGACGACTTCGTCACCTACATCCAATACATGCTCCAGATCGGCGGCGACTGGACCTGGAAGGACTGGAACCCGGACCTCGTGGCTCTCTCCGACAAGATCAACCCCGGAAACGCCACCGCCGACGACTTCGACATCTCCCCCTTCTACAAGAAGGGCGGCAAGCTCCTCCACTACCACGGTTACTCCGACCCGTCCATCGCAACCGGCTCCTCCGTCTACCTGTACAACCACATCCAGGAAGCCCTCCGCCCCCAGGACATTCCCATCGACGACTTCTACCGCTTCTTCCTGATCCCCGGTATGGAGCACTGCACCGGCACGCCCTCCGACCAGGACGCTCCCTACTACATGAACGGAGACAGCCAGGCCGCTTCTCTCTCCGGAACCGTGTTCGGTGTGCCCGGCTTTAATGACCCCAAGCACGACCTCGTCCTGGCTATCATGAACTGGGTCGAGAACGGCACGGCCCCTGACTATCTCATCCCGACCAAGTTCAAGAACGATGATGTCGCTGATGGCGTCGACAAGCAGAGACCTATTTGCCCTCATCCCCAGCTGGCCAAGTACAAGGGCTCTGGTGATGTGGACAAGGCTGAGAACTGGTACTGCGGTACTCTTTACTAG |
| An3040-1 | ATGGCAGATGGCGGCGGCGTCCGCGGTTACTCCATGCTCATCATCCTCCAAGAACTCATGTACCGCGTCTATGTCGAATGCGAAGGCAAAGCCCCTCGTCGCGATGAAATCCCCAAACCCTGTGACCACTTCGACCTCATCGTCGGCACCGGAACCGGCGGCCTGATCGCCCTCATGCTGGGTCGGCTACGACTGGATCTCGAGACCTGCAAAGATGTCTATGTCCGCATGACCCGCCGCGTGTTCGAAACCGACAAGACCTTCGCCGGCATCCCATTTCATAAAACACTATTCAAGGCTTCCAAGCTGGAAGAAGCCATCCGTGAATGTGTCCGCGAACATACCGTTTTTGAAGCGGAGGGGAACGATATGAGTCCGTCGGCGCGCAATTCGCTGGCCAGTGCTCCTTTTAGTCCCAACTCCATGTCAGTGCCCCAGCGCTCGGGCAGTCGGGCTAGCTTCAGTACTACTACCTCCCATTCGTCGGGTCATGCTAGTCAGCGCAATTCGACGTTTGTGAACGGCTTACGCTGGGGAAATCCGGATGCGCTGTTGTATGATAATCGAGAGTATCGGACGAAAACTGCTGTGACTGCACTCTACAAAGGTACCACGTCCCGCAACGGCTCGACCGTCCTCCTCCGGTCCTACGATTCGCGCAAAGAGCCCCCGCCCGAGTTTAACTGCACAGTCTGGCAGGCTGGTCGTGCCACTTCTGCCACCGGGCTAGCCTTCAAGCCCATTCAGATTGGACAGCACGTCTTCATCGACGAAGGTGCCGGCACCTACAACCCCTCGCCGCAGGCGCTGGATGAAGCGGTCATGAACGAGTGGCCCGGTCGCGAGATCGGAGTGTTTGTCAGCGTGGGAACTGGCAAGCGCCCACCAGGCACCAACAATCGGCAACACGAATGGTGGGAGGACTTCTTTGGGGATGCCCTGGGCACGTTCGCGGAAGCTCGTCGACGCCTGATCGCCAAGATCGAGGGGTGCGAGGATATCCATTTGGCGATGCTGCGCGACCATCTAGCTAAGCGCAATGTGAGCAAGGACAACTACTACCGGTTGAACGTCGAGGTGGGTGTGGGCGAGTTTGGAATGAATGAATGGAACCGGCTGGCAGACATCAGCACCAACACACGACGCTACCTGACGCGACCGGAGGTGAAGCACCAGATCCTGGACGCAGGTGTCAAGTTCGCCAAGATTGAGCGCCAACACCGCCGGCTGGCTGACCATGCGGCGGCGGCAGGTCAGGTCGATGATGGGACCTCGTCCATCATGCACAGTCCAGTGCTGTCGGTGCCGCCGCCATCGCACCCGATGGCCGTGGAGCTACCGGCTGAGCTGCCGGGTGACTTTGTGCCTTATATCACAACAGAGGACTCGCTGCCGGCGCACCCCACGCCACAGGACGCCGTTCTGTCATCACCGGGGCGGACATCAGGCGACTTGGCCAGTCCGGGGGCTGGCGATGTGAGTCGGCCCAACTCGCGGGCGCATGGGTCCTCTCGACCCAGCACCGGGCATGGCCATGATGGGATGCCTCCGCCTGTGCCGCCCAAGACGCCGATTCCCTATCCTTCCGAGTATCCTAGTGAACTGGGAGGGATTCCCATGCCGATGCCGACGACGACCAGCCCGGGACATAGTCACAATGGAAGTTTGAGTGGGAAGATTCGACCGCCGTATCCAGTGGACGAGCCACCGGTGGTAAACAAACAGCGGAAGCCGAGTTACCATGTGCGGTCATGA |
| An3040-3 | ATGGATTACTCCGTCAACCTCCGCCGCAAGGATACCACCAAGGGTCCGCCCTTGCGGATCCTGTCGCTTGATGGCGGCGGCGTCCGCGGTTACTCCATGCTCATCATCCTCCAAGAACTCATGTACCGCGTCTATGTCGAATGCGAAGGCAAAGCCCCTCGTCGCGATGAAATCCCCAAACCCTGTGACCACTTCGACCTCATCGTCGGCACCGGAACCGGCGGCCTGATCGCCCTCATGCTGGGTCGGCTACGACTGGATCTCGAGACCTGCAAAGATGTCTATGTCCGCATGACCCGCCGCGTGTTCGAAACCGACAAGACCTTCGCCGGCATCCCATTTCATAAAACACTATTCAAGGCTTCCAAGCTGGAAGAAGCCATCCGTGAATGTGTCCGCGAACATACCGTTTTTGAAGCGGAGGGGAACGATATGAGTCCGTCGGCGCGCAATTCGCTGGCCAGTGCTCCTTTTAGTCCCAACTCCATGTCAGTGCCCCAGCGCTCGGGCAGTCGGGCTAGCTTCAGTACTACTACCTCCCATTCGTCGGGTCATGCTAGTCAGCGCAATTCGACGTTTGTGAACGGCTTACGCTGGGGAAATCCGGATGCGCTGTTGTATGATAATCGAGAGTATCGGACGAAAACTGCTGTGACTGCACTCTACAAAGGTACCACGTCCCGCAACGGCTCGACCGTCCTCCTCCGGTCCTACGATTCGCGCAAAGAGCCCCCGCCCGAGTTTAACTGCACAGTCTGGCAGGCTGGTCGTGCCACTTCTGCCACCGGGCTAGCCTTCAAGCCCATTCAGATTGGACAGCACGTCTTCATCGACGAAGGTGCCGGCACCTACAACCCCTCGCCGCAGGCGCTGGATGAAGCGGTCATGAACGAGTGGCCCGGTCGCGAGATCGGAGTGTTTGTCAGCGTGGGAACTGGCAAGCGCCCACCAGGCACCAACAATCGGCAACACGAATGGTGGGAGGACTTCTTTGGGGATGCCCTGGGCACGTTCGCGGAAGCTCGTCGACGCCTGATCGCCAAGATCGAGGGGTGCGAGGATATCCATTTGGCGATGCTGCGCGACCATCTAGCTAAGCGCAATGTGAGCAAGGACAACTACTATCGGTTGAACGTCGAGGTGGGTGTGGGCGAGTTTGGAATGAATGAATGGAACCGGCTGGCAGACATCAGCACCAACACACGACGCTACCTGACGCGACCGGAGGTGAAGCACCAGATCCTGGACGCAGGTGTCAAGTTCGCCAAGATTGAGCGCCAACACCGCCGGCTGGCTGACCATGCGGCGGCGGCAGGTCAGGTCGATGATGGGACCTCGTCCATCATGCACAGTCCAGTGCTGTCGGTGCCGCCGCCATCGCACCCGATGGCCGTGGAGCTACCGGCTGAGCTGCCGGGTGACTTTGTGCCTTATATCACAACAGAGGACTCGCTGCCGGCGCACCCCACGCCACAGGACGCCGTTCTGCCATCACCGGGGCGGACATCAGGCGACTTGGCCAGTCCGGGGGCTGGCGATGTGAGTCGGCCCAACTCGCGGGCGCATGGGTCCTCTCGACCCAGCACCGGGCATGGCCATGATGGGATGCCTCCGCCTGTGCCGCCCAAGACGCCGATTCCCTATCCTTCCGAGTATCCTAGTGAACTGGGAGGGATTCCCATGCCGATGCCGACGACGACCAGCCCGGGACATAGTCACAATGGAAGTTTGAGTGGGAAGATTCGACCGCCGTATCCAGTGGACGAGCCACCGGTGGTAAACAAACAGCGGAAGCCGAGTTACCATGTACGGTCATGA |
| An3131 | ATGTCTGCGCTTAAAGACGAAAAGACCTTGAGTGAGCTACGGCCCCGGGTCCTTATCCAACCACTTCACAACAGCATCCCGTCGGAGTTGCTGCCTCGGTTCGACCCGGTCTATGTGGAACACTACAACAAATACAATGCTGGGAGGCTACATACGCATGAGGTTCCTATCGAAGACTTCCGCAAGAACCCAGCCAAGTATGCCATTGCCTACGGCCGTGCTCAAGGTCCTGATGTCTTCCGCATCACGGAGCAGAAATGTCCCGTGCAAGGGGGCGAGATAACCATCCGTATCTTCGAGCCTGCCCCGAAAGCGGATGAGCATGGCAAGGCCAAAAAGAGGGCTGCGTTTGTCAACTTCCATGGGGGAGGCTGGGTGTTCGGCGATCTCTCAGTTGATCACGATTTCTGCAAGACACTCGTCGATGGCCTGGACGGGCACTTGGTCGCGTTTGATGTCGACTACCGGCTAGCTCCTGAGCACAAGTACCCGATCCCCGTTGACGACTGCTGGACCGCTTTCAATTGGATCCGCTCCCAGAAAGCAGAGGAGTTCAACGTTGACCCGAATCGAATAGCTGTTGGAGGTTGTTCGGCCGGAGGCCACCTGTCAGCCGTGGTCGCTCATCTCTGCCGTAATGGCGGCATTCCGTTGCGCCTGCAGGTGCTGAACGTGCCCGTATGTGATCTACATAGCGGCTACACTCCGGATGGTGAATTCGATCGGGAGAACTGTCCCTATGAGTCCTACAGAGAGATGGAGTTCACCGCAGCTCTTCCGGTAGCACGGATGGCTTATTTCCATCGACACTTTTTGGGGGTTCCCCGGCCAGCACGTTCAGAAGAGGACTGGAAGATCTCCCCCATATTTGCGCCTGACTTTTCTGGACTAGCACCTGCGTTGGTCTTCACCGCCGAAATGGATCCTCTGCGGGACGAAGGGGAGGCCTACGCTGCCAAATTGAAAGCTGGCGGTTGTCGAGTGGAAATGATGCGTATGGCAGGAGCACCCCACACATTTGCCATGTTGGATGGCATCTTAGAGAGCGGCCGTATATATACCGAGAAGGTCATCGAAGCGATGAAACGGGAACTAACAGGGTAA |
| An3196 | ATGGGGATAAAGCCAAAGTGGCTACGAGATATTGCGTCTCTGGTTTTCTCTGGGTATTATCTTATCGCAGCCGAACAGGCAGATGAGAAGGTTCGCCGGGTCCGTGCTACGCTCACAGTAGAGCATATGCGTGTGTCGTGGAATAAGAGCACCACACCGTATCTCTGGGCACTCGCTAAATTGGGCCGCCCGCGGCTCACGAAGTACCCTCCGCGTGCCATTCGCATCCCCCGACCAGCGTCCTCAATATACAAGGAGCCAACAAATGCGTGGCTTTACTTTGATGGGTCATTGGCCGCGCTACGTGATCAAACATGCATTGTCCTGGATATTCCTGGAGGTGGATTTGTCTCGATGAGCCCGCGGCATTCCGAAGACCGACTTCTGGCTTGGGCAGCGAGAACCAAAGTTCCCGTACTGAGTCTCGACTACAAAAAAGCACCGGAGTATCCGTATCCGTATGCTTTGAATGAATGTTATGATGTATATCACTCCATTGTCTCCACCCGTGGGCGGTGCTTGGGCCTCAACGGACGGACTCGTCCTCGTGTCGTCATCACTGGTGATAGTGCTGGTGGTAACCTCGCTACTGGCGTCACCCTTATGATCCTTCAATCTGGCAGTACACATGCCTCTAGATGGCAAGGACAAAACATGCTTCCGCCTCCGGATGGACTAGTTCTACCATATCCCGCGCTGAACATGAAGATAGAAAGCTGGATGACGGAAGAGCAAATGGCTTTAATACAAGAGAAGAGCACACGTCGAACCAATGAGAGCGTGCTTCGAAGAAAAGATATGGACTATCACCGGTTGACGCCTTTTACCTCTCCGGGAACGTCATTCGGAGATTTCCAGCGTGACTCGTTCTCTGACGCGGATCTGGAAGTCGGAAATTTGGTTGACGCTACCTCGGAGAAGTTGGCACAGCAACAGAAGATGGACGTCACTGAAGAGGAGGTGGCTGCCATCACTGAGCACCAGCCTAAGCAGATCCGAACACGACTGGCTGTTTCATCGATGATATCGTATGTTAATGATCGTATATTAACCCCCGAGATGATGAGGGCAATGATCATCCTTTACATCGGACCGCACAACCGCCCCGACTTCAATACGGACTACCTACTCTCCCCAGCCCTGGCTCCGGAAGAGCTCTTAGCACGTTTCCCCAAGACGTACATCATCACCGGAGAACGTGACCCTCTAGTTGACGACACGGTCATCTTCGCCGGGCGACTAAGACAAGCTAAGCTCCGCCAATTCCAGGAACGACAAGAACTGGGCCTCGAAAAGTCTTATCGCAGGTTCAACGCAAAGGACCATGTCGAGGTTTCCCTATTACCCGGGATCTCTCACGGGTTCATGCAAATGGCCGGCTTCTTCCCCGATAGCTGGAAGCACATCTACCGCTGCGCAACTTGGATTCAAGATCTATTCGACCTCGCCCAGACTAAGAAATCATCTTCTGGCCACCTGAGGGCCCTCACCAACGGCGCACAAATGCCAAAGGGGCCCAAGTCCGTTCCCCGCGGCAATAGCTCCCGCAATCATAGACGTCGTCTCACGGGAGAGTCATCCGGCGACGAGGATAGGCCTCTCGAGATAGGCATCAAGAAACTGACACCACTGACACCATTGCATCGCAGCTCCAGCGCACCCATCGACAGTACCGAGATTGATAGTCAAGATATCGCTGCCAAGGATGGCGACAGCGAGGATGCTAAAAAGGAGCGCATCCGTAGCGTCTCCCGAGGCCGAACTCTGCGGCCAGGGATCACCAGGAAACAGCGTCCCATTCCAATGAGGTTAACGATCCCTCCTCGAGACGGGTATATCTCTGGATCGCCGAGTCCTATTCCTTTGCGGGAAAGAAACCGGAGTATTAACAGTCTTGCCAGCGAGGAGGACCTGCTTGACCGGCGTATGAATGGCTTAGCTGGAGGGTTGATGGGCATTGGCGAAGGTGCTAGGACGCCTTAG |
| An3559 | ATGGCCACTCTTAGAATGGGCGGCGTTGCCGCCGCAGTCACAGTGGCCATAGCACTCTTCGCCATATACCTCTCACCATCCACTGATGTCATAGTTGAACATATCAATGGTTACTACAATGTCGGTTTCCCTAAAGCACCCGTATCTCCCCCTACAGTTTATGACCCTCATCGTGGCATCAAATATATTGGCACTTTGTCAAATGGGGTAGAACACTTTCAGAACATCTTCTACGCAGAGGAGCCGACTGGCCCCCGTCGCTTCGCTCCCCCAGTCCCCGTCCGCCCACCGAAAGGTTCTGTGATCAATGCCACTCAGTCTGGAGCATGGTGTCCACAGGGGACAGGAGATATCCTGCCTTTCACGAGTAAAGTGTCGAATATCAGTGAGAATTGCCTTAGCTTGAGGGTTGCTCGCGCGACCGGTGTGAAAACCCAGGATAAGCTTCCTGTTGTGGTGTGGTTACATGGAGGCGGCCATGCTCTGGGCTCTGCTTCTGACATTCTTTACAATCCAGAAGGACTGCTTCAAGAGGCAGTGGCAGCGGGGAAGCCGCTGATATACGTCGGGATAAACTACCGACTAGGATTCTTTGGATTCGCGACCAGCAAGGCTATGATTGCAAAGAAGCAGACAAATGCCGGACTGCGTGACCAGCGCGCTGCCTTGGAATGGGTCCGGGACAACATCGAAGCCTTTGGGGGTGATCCCAGCAGAGTAACCGCTATCGGACAGAGCGTCGGGGCCAGCGACATCGGCCTGCATTTGACATCATTCAACGGCACCAAAGGTGTCCCCTTCCGACGCGCAATCATGATGTCTGGCGCCCCAGGTGTCAACTTCAACAGTGACCCAAGTCTGGTATCCGAGAACACCGCAAACATAGCGCGAGAGGTTGGTTGCATCAACACCAATGACGCCGAATCCCTCGAGACCCTCGAATGTCTTCAAAGCATCCCCTTTGACACCCTCACCAACCTCTCCGTCACGGCATCACGTGCAGCTCGCCCCCCGTTTGGAGAAGGATTTTTCTACCCGACCATTGACGGCGACTTTCTCCCCGCTCGACCCTCCGAGCTCCTGCGTGCCGGCAACTTCGTCAAAGATATCCCTATTATTGCTTCGTGGGTGGCCAACGATGGAGCCTGGTATGCCCCGCCCCCCACCTCCACCGACGAAGAAGTGCTTGCTAGCTTCGGACTGTGGCTGTCGAAGCTCTCCACTTCCACAAAAGCAAGACTGCTGGATTTGTATCCCATCACAGACTTCGCGCATATGATCCGCGAGAGCTACGATGGCCCTATCTCACCGCAGTACTACCGCGCCGCGCAAATGAATCGTGATATCTGGTTTACTTGTCCAGTGCTTGATTTTGCATGGCAGTACTTCCAAAAAGGGGGGTCTGCCTCATCTAATATATGGCTCTACGAGCATAATGCGACCCGATACGCACCAGTATTTGAAGTTATGGGCGTACCGATGTGGCGCGTTGCCCATCTTAGTGATATTCCTTATGTCATGAATAATGCTAATTTGGAGGGTGGGGCTGATAACTCCCCGGAACAAATGAAATTGGCACGGGAAGTGAGCAGACAGGTAATTGGGTTTGTGCATGATGCGAGTCCTAGTGAGGCGTGGCCTGCGGCATTCTTTGGGATCAGTGAGCAGGGCCTTTCGAAAGATTCACCTGATCAGCTGTCGATCCAGACTTTCGGGGGCCCTATGGGATCGCGAGCCGTCACAGTCCGCAAAGGTGCAGATACGAGTGGTTCCACAGAAGCAGAAAAGGCAGTGTTATGGGAGAGGCTATTTGACCGGTGCGAATTCATTAATAGTGCGCAGATGCGAGCAGAGGCCGGAGTATAG |
| An3936 | ATGCAGCTTCAATTCATCGTGGGCTCTTTGCTTCTCTTTGGCTCTGCTTCGGCAGCCCCAGCTCCGCCAGCAGATGCAAGATCCCACCCCAGCATTGATTCTCTGGGTACCCTCCAAGCTCTGAAGTATAACAACTTGGGCCCAGGAAACAACGGCACCGCCGCAGTCTTGGTCTACGATCGACTGCCATACCTCAGCGCAGAATCTAGGTGTGCATCCATTGGCGAAGCCCTCTATCCGCTGCAGGATGTATCACAAGCCAACGCCACAGAGATAGGGTACCAGCTCGACTATCTGGTTTACGCGAAAGATGTGGCGGCAAATAGCTCCTTTTGGATAGCCAAGGGCTCACCGAATGGCTGCCAGGCATACTCTCACAGCCGTAAGCAAGTCATTCCCGCTCCATGTGATCGACAACTCCCTGCCCTTTGCACCTCCAGCGTCCCTCCCACTACCGACAAGGACAGGGAAGCCGTGAACAAGTCCAAGATCTCTATCTCATTCGATGATTACCGTATGACTGGCTATCGTGATGGGCGCTCGTTTCGGTTCCTAGGCATTCCCTTCGCAGACCCGCCTGTAAGGGATTTGCGATTCGCACCCCCGCGCCCCTACTCAGGTCCCAAGAAAATTGACGCGACTAAAATGGCGGACTCATGCATTCAATCGGTTTCGGGCTTTGGAACACTCGACAATGGGGGTATCTCGGAGGACTGTCTATACCTGAACGTCTATTCTCCTGTCCTGCCCTCTTCACATGACAGGAACTCCACCCGCAAGCCTGTCGCGGTATACTTCTACGGTGGTGCGTTCACCAGTGGAACGGCGTCCATGGTTGATTACGATGGAGGGAATTTCGCCAGCCGTAACGACGTTGTTGTTGTAACAGTCAACTACCGAGTCGGTGCTCTAGGGTGGCTGACCACGGGCAACCTCACCACCGGCAACTATGGTACACGCGACCAGATACTTGCATTGAAATGGGTCAACAAGTATATCGAAGCATTTGGTGGAGATCCAAACCATGTCACAATCTTTGGTCAATCCGCCGGAGGTCAGAGCGTGATTGCGTTGCTCTCTTCGACTGCAGCCCACGGCCTATTCTCTGGTGCCATTGTGCAGTCCGCCCCCGTAGACCTTCCCTGGTTCGCACGTGAAGTTTATACCAAGATTGTTACTCCCAACGTCGCGGGGGCTGTTGGCTGCAACGGCACCACATCGGAAACCGCCCTCATCTCCTGCCTGAGATCAGTTCCGGCAACCCGGTACCTGGACAACACTACTGAGTTCGAAGCTGCCATGACAGCCTCCACCGAGACCATCGCCAGCGATTGGCTGCATTCATCTGAGATCCTCGCATCGATCGAGCCCCTGATGCCCATAGTAGATGACATCGGCAGTGGCGTCATTGACGACCAGTTCTACCGTCTTCTTGCTTCGGACAGGCTACCCAACCGCGTTCCAACCATGTTCACAACAGTCACTACCGAGGCTGCTCTCTACGTTGATCAGTATGTCCCGAACCTTGGGGCAAGCGAAGCTGCTCTTGAGCTCGTCTACAGCTATGCCTATCCCACGTCACTCATCAAGTCCCTCATCGCCACCGACGCCTTTCCTCTGAACGCCTCGGACTCTGACAGTGTCCGGAATACTGTTGCGGACGCGCTCACCCACAGCGAGTGGAGCTGTCCTCAGGCATATCTCCTCCGCCATGGCGGCCGACATGCGTTCCCGCGCCTCTGGGAAGTTGAAGTCAGACACGGTCACGTCCAAACCACCGTGGATGTCCCCAGCATCTGCTCCCCCAACACTGATTTCAACGCCACGTGCCACTCCGCAGATGTGCTTCCTGCCTGGGGCACTCTCAACAGCAAGACGAAGAATGTATCCCCATACTACAACAAACGGGACATCCTGCATTCCCGTCTCCTCAACGACATCTTCGGTTCATTCTTCCGCACCCGCAATCCCAACCCCGATCTGGAGATGCTGAAGCTTCGTGGGCCCGCCTATGCATCGACTTATCAGATCTTCGGTCCTCGCGGATACTACATGCCCGAGTATGAAATCGCGGAGCGAAATGTGAGTGTTCTGGACATGCCGCCGTCGATTGTGGCGAATCCAGGTGTTACGGAGAAGTGTGCCGTGTTCGAAGACTTTGGGTTCAGCTTCCAGAGAGCCAATCTTACTGTCTAA |
| An5004 | ATGATTCGGCTTTTGCTACACACCGTTATTTTCCTATTTGTCGGCCTCAGTCGAGCAGCAATCGTCCCCCTTCCTCCAAGCACCGGACCATGCGACGTCACGCTCCAGGCGAGTGAACTGGTCGATCAAGCTCGTACCAATCCATTCGATTCAAAAGGGGGTAAACGCGCTCTAATGGTCACGACCTTTACACCCGTCAATTGCGGAAGTGTGCTCTCTGAATTTTACATACCGAATGCAACAGCAGCATACGAGGATGAGAGTTTCCAGTCTCTTGGACTAGCCGCAGGGACTTTCGAGTCCTTTCGTATCCAGACACAGCAGCAGCCATCACCTTCAATTGTTAACAAAAGCTATCCAATTGTGCTCTTCTCACCAGCTCTGGGAACATCAAGATTGGTGTACACCTCGCTGCTTCAAGACATTGCCAGTTACGGATTTGCTGTCATTTCCGTCGATCATCCATACGATGCCAACATTGTGGAATTTCCCGATGGCCGTACGGTTATCGGCGTTCTCGAAAACACCACTACAGATGCGCAGTTCACTTGGGCTATGAACGTTCGCGTCCAAGACATGATATTTGTTTACAATCAGACTCGCAATGAGACAGCAGTCAGAGACATATTCCCCTTGTCACTGAAAAATCCACATCTGCTATCTCTCGACCGAGTCGCAATTACTGGCCATTCCCTTGGCGGAGCAACAGCAGCGCAGACAATGTTGGTTGACAACCGATTTGTCGGCGGTATCAATTTAGATGGTACATTCTGGGGATCTGTCCTCACTAAAGGACTGTCCAGCCCTTTTCTGCTTTTCAGTAATGCCAACCACACAGCGGCAACAGATCCTAGCTGGGGAACATTCTGGTCGAATTTACGAGGATGGAGATTGGAGCTACGACTCGCACAGTCTAAGCACTATACCTTTTCCGACTTTCCGGTGCTTCTCGACTCCCTGAGTATCTCTGACGAACTGAAAGAAATTGTCCAGGCCAGTTATATTGGGACGATTGGTGCTTTGCGCGCTAAGAACGTGATTGTCTCGTACATCGTTGCTGCGTTGCAGTACTTTGCTTATGGGCGCACGTCAGATCTACTGAGTGGCCCTTCGGCAGCATATCCTGATGTTACTTTTGTACATTCTGCCTAG |
| An5100 | ATGGCGTCCCACTATCTCAGCACGTCGCCGGATACCAACATCCATTTCACCATTACTAAAGCACAGTCTCCATCTTCACCTACGCCCCTTCTTCTCTTTCTCCACTACTGGGGTGGTTCGAGCGCAACATGGTACAAGCAAACCTCACCTACTTCCCCACACACCTTGAACAACATATACAACACTGTTACAGCTGATCTTCGCGGCTGGGGTCAGTCCACAGGACCTGCAGACTCCGGCGCCAGCTCAAAAGACTACTCCATTACGCCCATGGCATCTGATATCGTCTCCATGCTATCGCACCTCCAAAGCACCACCTCCCTCCTCGACAACGGTGTGATCCTAGTCGGTCACTCCATGGGAGCAAAGGTTACACTGGCTACTCTCTCCAAGCTTTCCGACAATCAATTATCCCTCGTGAAGGGCCTCGTGCTTGTCGCTCCGGCGCCCCCAACTCCATTGGTACTCCCTGCTGAGATGAGCGAACAGCAACGCAAAGCGTATGATAACGAAGGCTCCGTTCGGTGGACGGTCGAGAACGTTCTGTCCAGCGTACAGAATATTTCGGGTTACGATATGGAGTTGGTCGTCAAGAATAGTCTGGCAGGGAGTACTCTTGCTCGGGACGGCTGGATTCTACATGGTATGCAAGAAGATATTACATCTGCTTTAGATGAGGTGTCTACTCAGCTTGAGGGCCGGAAGGTTAAGGTGGGTGTGCTTGCTGGGGCGGATGATATTGTTGAGAACAAGGATCGGGTCGAGAAAGAGGTTGCTGGGGCGCTTACCCAAAGAGGCTTTGAGGTGCAGTTTGAGGTACTAGGGGGCGTGAAGCATTTGATTCCGTTGGAGAGCCCGGAGAGCGTGGCTAGAGCGATTCGTTCGATTGTTGAATAA |

**Table S3.** The binding energies of the ligands and the receptor proteins An286, An1556-4, An2017, An2588, An3559 and An5004, and the amino acid residues formed hydrogen bonds with the distances.

| **Enzyme** | **Parameter** | **Caproic acid** | **Ethyl caproate** | **Caprylic acid** | **Ethyl caprylate** | **Capric acid** | **Ethyl caprate** |
| --- | --- | --- | --- | --- | --- | --- | --- |
| An286 | Binding energy (kcal/mol) | -3.15 | -3.79 | -3.30 | -4.05 | -3.59 | -3.36 |
|  | Residue formed hydrogen bond (the distance, Å) | Gly117 (2.6)  Ser190 (2.7)  Ser190 (3.0)  His326 (2.0) | Ser190 (2.7)  His326 (2.1) | Gly117 (2.5)  Ser190 (2.6)  Ser190 (2.8)  His326 (2.0) | Gly117 (2.5)  Ser190 (2.7)  His326 (2.3) | Gly117 (2.7)  Ser190 (2.6)  Ser190 (2.9)  His326 (1.7) | Gly117 (2.4)  Asp189 (3.3)  Ser190 (1.9)  His326 (2.0) |
| An1556-4 | Binding energy (kcal/mol) | -2.71 | -3.46 | -2.81 | -3.40 | -2.57 | -3.22 |
|  | Residue formed hydrogen bond (the distance, Å) | Gly128 (2.3)  Ser210 (2.7) | Gly128 (2.2)  Ser210 (2.6) | Gly128 (2.4)  Ser210 (2.5) | Gly128 (2.2)  Ser210 (2.6) | Gly128 (2.1)  Ser210 (2.7)  Ser210 (3.4) | Gly128 (2.3)  Ser210 (2.6) |
| An2017 | Binding energy (kcal/mol) | -3.49 | -3.53 | -3.47 | -2.68 | -2.69 | -3.04 |
|  | Residue formed hydrogen bond (the distance, Å) | Arg157 (1.9)  Asn455 (2.4)  Ala478 (1.8)  Thr560 (2.6) | Gly154 (2.4)  Gly155 (2.1)  Ser196 (2.8)  Ser196 (1.9)  Asp448 (2.8) | Arg157 (2.0)  Asn455 (2.5)  Ala478 (1.7)  Thr560 (2.6) | Gly154 (2.7)  Gly155 (2.4)  Ser196 (2.7)  Asn455 (3.2) | Arg157 (1.9)  Asn455 (2.5)  Ala478 (1.7)  Thr560 (2.6) | Gly154 (2.5)  Gly155 (2.2)  Ser196 (3.1)  Ser196 (2.2)  Asp448 (3.2) |
| An2588 | Binding energy (kcal/mol) | -2.84 | -3.59 | -3.18 | -4.33 | -3.37 | -4.62 |
|  | Residue formed hydrogen bond (the distance, Å) | Gly106 (2.3)  Ala107 (1.7)  Ser189 (2.5)  Ala190 (2.0) | Gly106 (2.0)  Ala107 (1.6)  Ser189 (2.5)  Ala190 (2.5)  His399 (2.6) | Gly106 (2.0)  Ala107 (1.8)  Ser189 (2.5)  Ser189 (3.0)  His399 (2.6) | Gly106 (2.1)  Ala107 (1.9)  Ser189 (2.6)  His399 (2.3) | Gly106 (2.1)  Ala107 (1.9)  Ser189 (2.7)  Ser189 (2.8)  His399 (2.2) | Gly106 (1.9)  Ala107 (2.0)  Ser189 (2.0)  Ser189 (2.6)  His399 (2.1) |
| An3559 | Binding energy (kcal/mol) | -3.75 | -3.95 | -3.95 | -4.33 | -4.40 | -4.09 |
|  | Residue formed hydrogen bond (the distance, Å) | Ser246 (2.8)  Arg496 (1.8)  Arg496 (2.0)  Arg496 (2.7) | Arg496 (2.1) | Ser246 (2.7)  Arg496 (1.8) | Phe339 (3.3)  Arg496 (1.9) | Ser246 (2.6)  Arg496 (1.9)  Arg496 (2.7) | Ser246 (3.1)  Arg496 (1.8) |
| An5004 | Binding energy (kcal/mol) | -1.73 | -2.94 | -1.95 | -3.58 | -1.79 | -3.50 |
|  | Residue formed hydrogen bond (the distance, Å) | Ser231 (2.7)  Asp254 (2.6)  Thr256 (3.0)  Thr256 (3.2) | Ser231 (3.1)  Leu232 (3.0)  Thr256 (2.8)  Thr256 (3.3)  Trp258 (3.3) | Ser125 (3.0)  Ser125 (3.0)  Ser125 (3.5)  Thr228 (3.4)  His230 (2.7) | Ser231 (2.9)  Leu232 (3.2)  Asp254 (2.9)  Thr256 (2.9)  Thr256 (3.4) | Thr130 (2.7)  Typ308 (3.2)  Typ308 (3.3) | Ser231 (2.9)  Asp254 (2.7)  Thr256 (2.9)  Thr256 (3.1) |
